# Endothelial and Astrocytic Support by Human Bone Marrow Stem Cell Grafts into Symptomatic ALS Mice towards Blood-Spinal Cord Barrier Repair

**DOI:** 10.1038/s41598-017-00993-0

**Published:** 2017-04-13

**Authors:** Svitlana Garbuzova-Davis, Crupa Kurien, Avery Thomson, Dimitri Falco, Sohaib Ahmad, Joseph Staffetti, George Steiner, Sophia Abraham, Greeshma James, Ajay Mahendrasah, Paul R. Sanberg, Cesario V. Borlongan

**Affiliations:** 1 Center of Excellence for Aging & Brain Repair, University of South Florida, Morsani College of Medicine, Tampa, Florida 33612 United States of America; 2 Department of Neurosurgery and Brain Repair, University of South Florida, Morsani College of Medicine, Tampa, Florida 33612 United States of America; 3 Department of Molecular Pharmacology and Physiology, University of South Florida, Morsani College of Medicine, Tampa, Florida 33612 United States of America; 4 Department of Pathology and Cell Biology, University of South Florida, Morsani College of Medicine, Tampa, Florida 33612 United States of America; 5 Department of Psychiatry, University of South Florida, Morsani College of Medicine, Tampa, Florida 33612 United States of America

## Abstract

Vascular pathology, including blood-CNS barrier (B-CNS-B) damage via endothelial cell (EC) degeneration, is a recently recognized hallmark of Amyotrophic Lateral Sclerosis (ALS) pathogenesis. B-CNS-B repair may be a new therapeutic approach for ALS. This study aimed to determine effects of transplanted unmodified human bone marrow CD34+ (hBM34+) cells into symptomatic G93A mice towards blood-spinal cord barrier (BSCB) repair. Thirteen weeks old G93A mice intravenously received one of three different doses of hBM34+ cells. Cell-treated, media-treated, and control mice were euthanized at 17 weeks of age. Immunohistochemical (anti-human vWF, CD45, GFAP, and Iba-1) and motor neuron histological analyses were performed in cervical and lumbar spinal cords. EB levels in spinal cord parenchyma determined capillary permeability. Transplanted hBM34+ cells improved behavioral disease outcomes and enhanced motor neuron survival, mainly in high-cell-dose mice. Transplanted cells differentiated into ECs and engrafted within numerous capillaries. Reduced astrogliosis, microgliosis, and enhanced perivascular end-feet astrocytes were also determined in spinal cords, mostly in high-cell-dose mice. These mice also showed significantly decreased parenchymal EB levels. EC differentiation, capillary engraftment, reduced capillary permeability, and re-established perivascular end-feet astrocytes in symptomatic ALS mice may represent BSCB repair processes, supporting hBM34+ cell transplantation as a future therapeutic strategy for ALS patients.

## Introduction

Amyotrophic lateral sclerosis (ALS) is a fatal disease characterized by gradual motor neuron degeneration in the brain and spinal cord leading to paralysis and fatality^1^. About 50% of patients die within 30 months of disease symptom onset and only 20% of patients survive 5 to 10 years after symptom onset^2^. Between 90–95% of ALS cases are sporadic (SALS) while the remaining 5–10% of cases are genetically linked or familial (FALS). Within FALS cases, several mutations in genes coding for Cu/Zn superoxide dismutase 1 (SOD1)^3, 4^, TARDBP (TDP-43)^5^, FUS/TLS^6, 7^, ANG^8^, and C90RF72^9, 10^ have been identified and are discussed in comprehensive reviews^11–13^. The clinical presentation and underlying pathology of SALS and FALS are similar, and treatment options for ALS patients are mainly supportive. The only FDA approved drug to treat ALS is riluzole, which extends the lifespan of ALS patients by only a few months^14^.

ALS is a complex multifactorial disease with numerous intrinsic and extrinsic factors underlying disease pathogenesis (reviewed in refs 15–24) such as glutamate excitotoxicity, mitochondrial dysfunction, oxidative stress, altered glial cell function, impaired axonal transport, protein aggregations, immune reactivity, neurotrophic factor deficits, and neuroinflammation. These multiple pathogenic effectors and the diffuse motor neuron degeneration in ALS present a formidable obstacle to treatment development for this disease.

Accumulating evidence has demonstrated impairment of the blood-CNS barrier (B-CNS-B) in ALS and this barrier damage likely represents an additional pathogenic mechanism. Compelling results showed structural and functional alterations in the blood-brain barrier (BBB) and blood-spinal cord barrier (BSCB) in ALS patients and in animal models of disease^25–33^. These studies demonstrated degeneration of endothelial cells (ECs) and astrocyte end-feet processes surrounding microvessels, impairment of endothelial transport system. Also, dysfunction of tight junction proteins has been implicated to compromise BBB/BSCB integrity. Additionally, vascular leakage, microhemorrhages, decreased capillary length and reduced blood flow have been shown in the spinal cords of ALS mice. Importantly, BSCB alterations were indicated in SOD1 mutant mice and rats prior to motor neuron degeneration and neuroinflammation^29–31^, suggesting vascular damage as an early ALS pathological event. These vascular pathologies, demonstrating impairment of neurovascular unit components in the brain and spinal cord, are key factors identifying ALS as a neurovascular disease^34^. It is possible that the initiating pathological trigger for ALS is a dysfunctional B-CNS-B, allowing detrimental factors from the systemic circulation to penetrate the CNS and foster motor neuron degeneration^35^.

Since the damaged capillary endothelium in ALS does not adequately maintain vascular homeostasis in the CNS, repairing the altered B-CNS-B by replacement of endothelial cells via cell administration may be a new therapeutic approach for this disease.

Bone marrow is a primary source of the putative EPCs but whether these progenitor cells are derived from hematopoietic stem cells or cells of endothelial lineage is still under debate (reviewed in refs 36–41). In terms of identifying the desired pro-angiogenic EPC lineage, it has been shown that EPCs are enriched in CD34^+^/CD45^-^ cell populations and are not derived from CD133^+^ or CD45^+^ cells^42^. CD34^+^ cells are pluripotent hematopoietic stem cells, capable of long-term *in vitro* self-renewal and of differentiation into multiple hematopoietic cell lineages that fully repopulate blood cells throughout adulthood^43, 44^. However, lineage potential of the hematopoietic progenitors during proliferation, commitment to multipotential differentiation, and maturation are controlled by various intrinsic properties^44^ and microenvironmental factors. Additionally, transplanted bone marrow-derived CD34^+^ cells migrate and home into damaged tissue, as shown in treatment of patients with ischemic or degenerative retinal conditions^45^ or cardiomyopathy^46^ by contributing to revascularization via formation of new blood vessels from existing vascularity in ischemic tissues.

Since EPCs are presumably derived from CD34^+^ cells, human bone marrow CD34^+^ (hBM34^+^) cells stand as a promising cell source for B-CNS-B restoration in ALS. We hypothesized that hBM34^+^ cells systemically transplanted into ALS mice induced BSCB repair by differentiating into endothelial cells. Whereas previous treatment-based studies have largely focused on pre-symptomatic ALS animal models, examining the effect of hBM34^+^ cells transplantation into a symptomatic mouse model of ALS increases the translational relevance to the clinic.

The aim of this study was to decipher the mechanisms of BSCB repair promoted by unmodified human bone marrow CD34^+^ cells intravenously transplanted into symptomatic G93A SOD1 mice. A specific focus was determining transplanted cell differentiation potential and dose effects on motor function and motor neuron survival in cell-treated animals.

## Methods

### Ethics Statement

All described procedures were approved by the Institutional Animal Care and Use Committee at USF and conducted in compliance with the *Guide for the Care and Use of Laboratory Animals*. All mice were housed in a temperature-controlled room (23 °C) and maintained on a 12:12 h dark: light cycle (lights on at 06:00 AM). Food and water were available *ad libitum*. Upon progression of neurological symptoms, a highly palatable liquid nutritional supplement was placed on the cage floor, ensuring access by the animal.

### Animals

All animals used in the study were obtained from The Jackson Laboratory, Bar Harbor, MA, USA. Seventy-two transgenic male B6SJL-Tg(SOD1*G93A)1Gur/J mice, over-expressing human SOD1 carrying the Gly93 → Ala mutation (G93A SOD1) at 7 weeks of age, were randomly assigned to one of four groups receiving different doses of hBM34^+^ cells or media: *Group 1* - hBM34+ (5 × 10^4^ cells/mouse, low dose, n = 15), *Group 2* - hBM34+ (5 × 10^5^ cells/mouse, mid dose, n = 15), *Group 3* **-** hBM34+ (1 × 10^6^ cells/mouse, high dose, n = 21), and *Group 4* **-** Media (n = 21). At 8 weeks of age and then weekly, mice underwent pre-transplant behavioral testing (extension reflex, grip strength test, and rotarod) and monitoring of body weight. When initial disease symptoms appeared such as deterioration of motor function and reduction in body weight (approximately 13 weeks of age), mice intravenously (iv, jugular vein) received either the appropriate hBM34^+^ cell dose or an equal volume of media. A non-transplant control group (*Group 5*), consisting of non-carrier mutant SOD1 gene mice from the background strain (control, n = 20), only underwent behavioral testing. At 14 weeks of age and weekly thereafter, mice again underwent behavioral testing until 17 weeks of age.

### Cell preparation and transplant procedure

Cryopreserved human bone marrow CD34+ cells (hBM34^+^, AllCells, Alameda, CA, USA) were thawed rapidly at 37 °C then transferred slowly with a pipette into a centrifuge tube containing 10 ml of Dulbecco’s Phosphate Buffered Saline 1X (DPBS), pH 7.4 (Mediatech, Inc., Manassas, VA, USA). The cells were centrifuged (200 g/10 min) at room temperature, the supernatant discarded and the process repeated. After the final wash, cell viability was assessed using the 0.4% trypan blue dye exclusion method before and after transplantation. Transplant cell concentrations were adjusted for each group: 250 cells/μl (5 × 10^4^ cells/200 μl/injection, Group 1), 2,500 cells/μl (5 × 10^5^ cells/200 μl/injection, Group 2), and 5,000 cells/μl (1 × 10^6^ cells/200 μl/injection, Group 3).

The hBM34+ cells were delivered intravenously via the jugular vein of mice under anesthesia with Isofluorane (2–5% at 2L O_2_/min) as we previously described^47, 48^ with minor modifications. Briefly, anesthetized animals received a sagittal incision at the base of the neck, and the jugular vein was identified. A 26-gauge needle was inserted into the jugular vein and needle placement verified by a reflux of blood into the syringe. A solution containing the cells was injected during 3 min, and immediately following injection moderate pressure was applied onto the needle vein entry point using a sterile cotton tip. After transplantation, the incision was closed and sutured using monofilament nylon (Ethilon) or a stainless steel wound clip. To assure hemostasis, needle puncture was performed through the muscle overlying the jugular vein, allowing the muscle, combined with digital pressure, to facilitate bleeding stoppage. The media-injected mice in Group 4 received 200 μl of DPBS, the same volume administered to the cell-transplanted mice. Animals in Groups 1–4 received cyclosporine A (CsA, 10 mg/kg ip) daily for the entire post-transplant period.

### Characteristics of disease progression

The evaluation of animal disease progression has been previously described^47–49^. All measures of animal disease progression were performed blind by independent investigators to avoid subjective bias. Body weight was assessed weekly throughout the study. Extension reflex, rotarod, and grip strength tests started on week 8 and were repeated weekly until 17 weeks of age.

#### Extension Reflex

The mouse was suspended by the tail and the extension of each hindlimb was observed. If the mouse showed normal hindlimb extension, a score of 2 was given. A score of 1 indicated partial hindlimb extension. If no extension was observed, the score was 0.

#### Grip strength test

The mouse was held by the tail and carefully placed with all 4 paws on the grid using an instrument to determine grip strength (IDTECH-BIOSEB, France). The animal was gently pulled by the tail and a sensor recorded the force (Newtons, N) with which the mice resisted the pull as a measure of muscle strength. The test was performed three times and the average of the tests was recorded.

#### Rotarod

The mouse was placed on a 3.2 cm diameter axle rotating at a speed of 16 rpm (Omnitech Rotoscan, Omnitech Electronics, OH, USA). The latency (seconds) that the mouse stayed on the rotating axle during a 3 minute maximum period was recorded.

### Perfusion and tissue preparation

All cell-treated, media-treated, and control mice were sacrificed at 17 weeks of age (corresponding to 4 weeks after initial treatment at symptomatic disease stage) for immunohistochemical analyses in the cervical and lumbar spinal cords for administered cell differentiation/engraftment and astrocyte expression. Histological analysis of surviving motor neurons was also performed in ventral horns of spinal cords. The mice (Group 3: n = 4; Group 4: n = 4) and controls (Group 5; n = 5) were injected with 2% Evans Blue dye (EB, Sigma-Aldrich, St. Louis, MO, USA) in saline solution (4 ml/kg body weight) via the tail vein 30 min prior to perfusion. Based on our previous studies showing that 1 × 10^6^ stem cell dose is beneficial in treatment of animal models of ALS^47^ and mucopolysaccharidosis type III B^50^, spinal cord EB extravasation was evaluated only in mice receiving the high (1 × 10^6^) cell dose vs. media and control animals. Mice were sacrificed under Euthasol® (0.22 ml/kg body weight) and perfused transcardially with 0.1 M phosphate buffer (PB, pH 7.2) followed by 4% paraformaldehyde (PFA) in PB solution under pressure control fluid delivery at 80–85 mm Hg to avoid capillary rupture. Mice assayed for EB extravasation received only the PB solution. After perfusion, the entire spinal cords were rapidly removed for the EB extravasation assay described below. In remaining mice, the cervical and lumbar spinal cord segments were removed, post-fixed intact in 4% PFA for 24–48 hrs, and then cryoprotected in 20% sucrose in 0.1 M PB overnight. Coronal spinal cord tissues were cut at 30 μm in a cryostat, every fifth section was thaw-mounted onto slides, and the tissue was stored at −20 °C for immunohistochemical and histological analyses.

### BSCB permeability

Evans Blue (EB) dye, 961 Da, was used as a tracer for assessing BSCB disruption. The EB extravasation assay was performed as previously described^51–53^. Briefly, after perfusion, mouse spinal cords were weighed and placed in 50% trichloroacetic acid solution (Sigma). Following homogenization and centrifugation, the supernatant was diluted with ethanol (1:3) and loaded into a 96 well-plate in triplicate. The dye was measured with a spectrofluorometer (Gemini EM Microplate Spectrofluorometer, Molecular Devices) at excitation of 620 nm and emission of 680 nm^54^. Calculations were based on external standards in the same solvent. The EB content in tissue was quantified from a linear standard curve derived from known amounts of the dye and was normalized to tissue weight (μg/g). All measurements were performed by two experimenters blinded to the experiment.

### Immunohistochemical staining of hBM34+ cells in the spinal cord

For identification of intravenously transplanted hBM34+ cell engraftment and differentiation potential, serial cervical and lumbar spinal tissue sections from randomly selected mice treated with different cell doses or media (n = 5/group) were stained with human anti-Von Willebrand Factor (vWF), an endothelial cell marker, and CD45, a hematopoietic common leukocyte marker. Briefly, the mouse monoclonal antibody (vWF, 1:100, Abcam, USA) was combined with the secondary antibody, monovalent goat anti-mouse Fab’ fragment conjugated to FITC (1:200; Jackson ImmunoResearch, USA), and incubated at room temperature (RT) for 2 hours. The tissue sections were pre-incubated with 1% normal human serum (NHS) and 0.5% Triton 100X in PBS for 30 min at RT and subsequently incubated with the previously prepared antibody cocktail overnight at 4 °C. Next day, slides were thoroughly washed in PBS and coverslipped with Vectashield containing DAPI (Vector Laboratories, USA). The tissues were then examined under epifluorescence using an Olympus BX60 microscope and images were taken for further analysis of vWF fluorescent immunoexpression.

In a separate set of the cervical and lumbar spinal cord tissue sections (n = 5/group), initial staining with the human-specific nuclei marker (HuNu) was performed as we described previously^47–49^. Briefly, the mouse monoclonal antibody (HuNu, 1:100, Chemicon, USA) was combined with the secondary antibody, monovalent goat anti-mouse Fab’ fragment conjugated to FITC (1:200; Jackson ImmunoResearch, USA), and incubated at room temperature (RT) for 2 hours. Prior to applying this antibody cocktail overnight at 4 °C, the tissue sections were incubated in blocking solution for 30 min at RT as described above. The next day, after several rinses in PBS, the tissues were placed in 10% normal goat serum (NGS) and 0.3% Triton 100X in PBS for 60 min at RT and then double-stained with mouse monoclonal antibody for CD45 (1: 200, BD Biosciences Pharmingen, USA) overnight at 4 °C. The next day, the slides were rinsed and goat anti-mouse secondary antibody conjugated to rhodamine (1:1000, Alexa, Molecular Probes, USA) was applied for 2 hrs at RT. After several rinses in PBS, the slides were coverslipped with Vectashield containing DAPI (Vector Laboratories, USA). The tissues were then examined under epifluorescence using an Olympus BX60 microscope.

To test for specificity of the immunostaining for vWF and CD45, the primary antibodies were omitted from control slides. No staining was observed in the control sections.

### Immunohistochemical staining of astrocytes in the spinal cord

Serial sections of the cervical and lumbar spinal cord from randomly selected cell-treated, media-injected, and control mice (n = 5/group) were rinsed in PBS to remove the freezing medium. The tissue sections were pre-incubated in a blocking solution of 10% NGS and 3% Triton 100X in PBS for 60 min at RT, followed by overnight incubation with rabbit polyclonal anti-glial fibrillary acidic protein primary antibody (GFAP, 1:500, Dako, Denmark) at 4 °C. On the next day, slides were rinsed in PBS and incubated with goat anti-rabbit secondary antibody conjugated to FITC (1:500, Alexa, Molecular Probes, USA) for 2 hrs at RT. After several rinses in PBS, the slides were coverslipped with Vectashield containing DAPI (Vector Laboratories, USA). The tissues were then examined under epifluorescence using an Olympus BX60 microscope and images were taken for further analysis of GFAP fluorescent immunoexpression.

### Immunohistochemical staining of microglia in the spinal cord

In a separate set of the cervical and lumbar spinal cord tissue sections from randomly selected cell-treated, media-injected, and control mice (n = 3/group), immunohistochemical staining for microglia was performed as we described previously^48, 49^ with minor modification. Briefly, tissue sections were rinsed in PBS to remove the freezing medium. The tissue sections were pre-incubated in a blocking solution of 4% NGS and 2% Triton 100X in PBS for 60 min at RT, followed by overnight incubation with rabbit polyclonal anti-ionized calcium binding adapter molecule-1 primary antibody (Iba-1, 1:500, Wako Chemicals, Richmond, VA, USA) at 4 °C. On the next day, slides were rinsed in PBS and incubated with goat anti-rabbit secondary antibody conjugated to rhodamine (1:500, Alexa, Molecular Probes, USA) for 2 hrs at RT. After several rinses in PBS, the slides were coverslipped with Vectashield containing DAPI (Vector Laboratories, USA). The tissues were then examined under epifluorescence using an Olympus BX60 microscope and images were taken for further analysis of Iba-1 fluorescent immunoexpression.

### vWF and astrocytic immunoexpression analyses in the spinal cord

Analyses of vWF and GFAP fluorescence immunoexpressions in the cervical and lumbar spinal cords from 17-week-old mice were performed in the ventral horns by an investigator blinded to the experiments. Animal codes were removed prior to analysis. Immunohistochemical image analyses for vWF and GFAP were performed by measuring intensity of fluorescent expression (%/mm2) in NIH ImageJ (version 1.46) software. Thresholds for detection of vWF and GFAP fluorescent expressions were adjusted for each image to eliminate background noise. To avoid bias in the analysis of fluorescent images, specific spinal cord areas were identified in a section using a 106/0.30 numerical aperture (NA) lens, and then areas of interest were photographed with either a 206/0.50 NA or 406/0.75 NA lens, photographing the slide in a random raster pattern. Briefly, measurements of cervical/lumbar ventral horn area were first performed by determining the cross-point of a line passing the central canal perpendicular to the midline. The area of ventral gray matter was determined below this line in the right and left cervical/lumbar spinal cords in coronal sections from each mouse group at predetermined uniform intervals (150 mm). For detection of vWF immunopositive cells in cell-treated mice, immunohistochemical images (n = 5 mice/group, n = 8–10 images/spinal cord segment) were taken in randomly selected areas from right and left ventral gray matter of the cervical and lumbar spinal cords at 40X. Fluorescent intensity (%/mm^2^) was measured in the entire image. Data are presented as averages of vWF cell immunoexpression of both sides. For GFAP intraparenchymal immunoexpressions in cell-treated, media-injected, and control mice, immunohistochemical images (n = 5 mice/group, n = 12–14 images/spinal cord segment) were taken from right and left ventral gray matter of the cervical and lumbar spinal cords at 10X. Density of astrocytes was determined as percentage per mm^2^ separately for the cervical and lumbar spinal cord sites as we previously described^49^. Also, fluorescent images of GFAP immunoexpression were taken of lateral and anterior white matter from both sides of the cervical/lumbar spinal cords at 10X for analyses. Additionally, GFAP perivascular immunoexpression was analyzed in the cervical/lumbar ventral horns of both sides from cell-treated, media-injected, and control mice. Fluorescent GFAP intensity of astrocytic end-feet (perivascular astrocytes) was measured adjacent to abluminal side of capillaries of approximately 25–30 µm in diameter (n = 4–5 mice/group, n = 14–16 capillaries/spinal cord segment). Density of perivascular GFAP immunoexpressions was determined as percentage per mm^2^ separately for cervical and lumbar spinal cord sites.

Also, the proportion of reactive versus non-reactive astrocytes was observed based on cell morphology. Reactive astrocytes present larger cell bodies and thicker, easily visible processes, as opposed to non-reactive cells, which have more delicate features.

### Histological staining and stereological count of motor neurons in the spinal cord

A separate set of cervical and lumbar spinal cord sections from randomly selected mice from each group (n = 5/group) were stained with 0.1% cresyl violet using a standard protocol for examination of motor neuron condition for the Nissl substance. Motor neuron numbers in the ventral horn of the cervical and lumbar spinal cords were determined by the optical fractionator method of unbiased stereological cell counting techniques using a Nikon Eclipse 600 microscope and quantified by using Stereo Investigator® software (MicroBrightField). The virtual grid (150 × 150 µm) and counting frame (75 × 75 µm) were optimized to count at least 200 cells per animal with error coefficients <0.07. Outlines of the anatomical structures were done using 10X/0.45 objective, and cell quantification was conducted using 40X/1.40 objective. The motor neuron numbers (20–25 μm diameter) were counted in discrete levels of the cervical (C1–C3, C4–C6, and C7–C8) and lumbar spinal (L1–L2, L3–L4, and L5–L6) cords (n = 7 sections/level/spinal cord segment/group separated by approximately 120 μm) and presented as averages per ventral horn for both spinal cord sides. Motor neuron morphologies were also analyzed in the cervical and lumbar spinal cords.

### Statistical analysis

Data are presented as means ± S.E.M. One-way ANOVA with Tukey’s Multiple Comparison test using GraphPad Prism software version 5 (GraphPad Software) was performed for statistical analysis. Significance was defined as p < 0.05.

## Results

The hBM34+ cells were intravenously administered into symptomatic G93A SOD1 mice (13 weeks old) at different doses and sacrificed at 17 weeks of age, corresponding to 4 weeks after initial treatment. Of the 64 total G93A SOD1 mice used in the study, five mice (Group 1 – one, Group 3 – three, Group 4 – one) were excluded due to death precipitated by conditions other than disease progression, more specifically, anesthetic complications during cell or media administrations. Also, four mice (Group 2 – one, Group 3 – one, Group 4 – two) were found dead at 16 or 17 weeks of age. Data on behavioral outcomes of these animals were included in analyses.

### Effect of hBM34+ cell transplantation at different doses on disease outcomes

**Figure 1:**
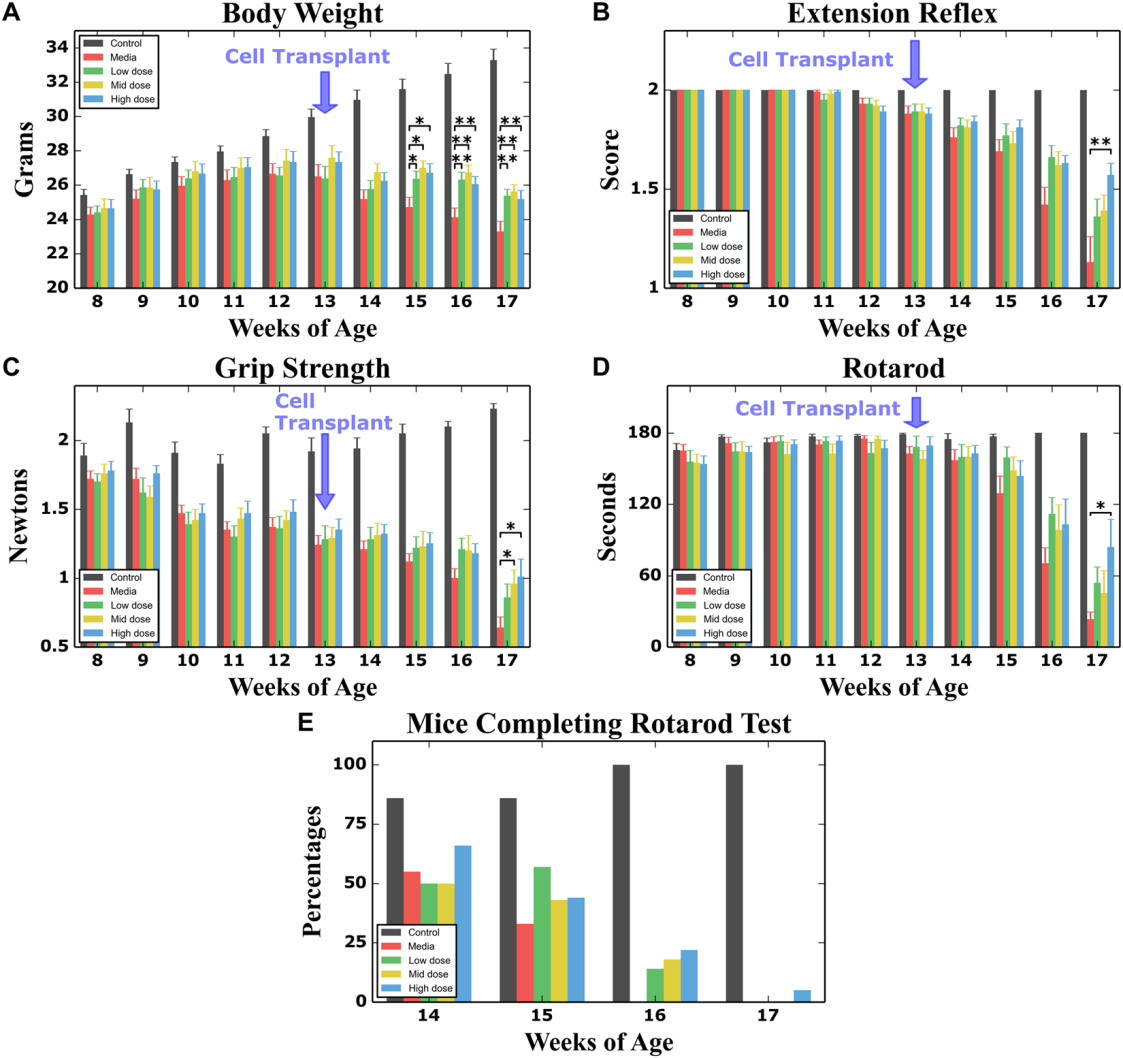
Characteristics of disease outcomes in symptomatic G93A mice receiving different doses of hBM34+ cells. All transplanted ALS mice during 4 wks post-treatment (**A**) significantly maintained body weight, (**B**) better extended hindlimbs, (**C**) delayed loss in muscle strength, and (**D**) stayed longer on rotarod vs. media-injected mice. More beneficial effects on motor function were determined in G93A mice treated with the 1 × 10^6^ cell dose, particularly at 17 wks of age. (**E**) Percentages of ALS mice transplanted with low (5 × 10^4^ cells), mid (5 × 10^5^ cells), or high (1 × 10^6^ cells) cell dose completing the rotarod test (180 sec) at 16 wks of age compared to 0% of media-injected mice, while at 17 wks of age, 6% of mice receiving the high cell dose were the only animals to complete this test. *p < 0.05, **p < 0.01.

Body weight, a general indicator of mouse health, and also a valuable marker for detecting progression of muscle atrophy, was measured weekly. As expected, body weight started to slowly decline at the symptomatic age of approximately 13–14 weeks in media-treated G93A mice and by 17 weeks of age, these mice had lost about 12% of their maximum body weight for this period. Although hBM34+ cell-treated animals lost weight more slowly, there were no significant differences between media and cell-treated mice at 1 week post-transplant. Significantly (p < 0.05) higher body weights were determined in all cell-treated mice at 2 weeks post-transplant (15 weeks of age) compared to media-injected mice (Fig. 1A). At 3 and 4 weeks after cell transplantation (16 and 17 weeks of age), all cell-treated mice maintained significantly higher body weights (p < 0.01) than media-injected mice. Of note, there were no significant differences in body weights between mice treated with different cell doses during the entire post-transplant period and at 17 weeks of age cell-treated mice weighed 2–2.5 grams more than media-injected mice.

Cell-treated mice also displayed superior performance in tests of functional ability. Deteriorating extension reflex was noted in media-treated G93A mice, beginning at 12–13 weeks of age (1.93 ± 0.03 and 1.88 ± 0.04 score, respectively), with extension progressively declining until 17 weeks of age (1.13 ± 0.13 score). However, hindlimb extension of mice treated with cells deteriorated more slowly than media-injected mice. At 16 weeks of age (3 weeks post-transplant), mice receiving cell transplantation displayed a tendency towards delayed deterioration of hindlimb extension compared to media-injected mice (Fig. 1B). At 17 weeks of age, only mice receiving 1 × 10^6^ cells showed significantly (p < 0.05) higher extension reflex scores vs. media-injected mice. Also, these mice demonstrated hindlimb extension scores (1.63 ± 0.04) superior to mice receiving 5 × 10^4^ cells (1.36 ± 0.09) or 5 × 10^5^ cells (1.39 ± 0.08).

In the grip strength test, media-treated G93A mice started to show decreased muscle strength at approximately 13 weeks of age (1,24 ± 0.07 N), with strength progressively declining during the course of disease until 17 weeks of age (0,64 ± 0.08 N) (Fig. 1C). Delayed loss in muscle strength was determined mainly in all cell-treated mice vs. media-injected at 15–16 weeks of age with significance (p < 0.05) in mice with 5 × 10^5^ cells (0,96 ± 0.10 N) and 1 × 10^6^ cells (1,01 ± 0.13 N) at 17 weeks of age (Fig. 1C). Although muscle strength in mice receiving 5 × 10^4^ cells (0.86 ± 0.10 N) was superior to media-injected mice at 17 weeks of age, no significant difference between these groups was determined.

Declines in performance on the rotarod test were observed in media-treated mice starting at week 13. Cell-treated mice demonstrated longer latencies for the entire post-transplant period vs. media-injected mice. At 17 weeks of age, ALS mice receiving 1 × 10^6^ cells showed significantly (p < 0.05) higher rotarod latency (84.07 ± 23.22 sec) compared to media-injected (23.28 ± 6.25 sec) or other cell-treated mice (5 × 10^4^ cells: 53.85 ± 13.66 sec; 5 × 10^5^ cells: 45.23 ± 19.12 sec) (Fig. 1D). Importantly, at 16 weeks of age, 14, 19, or 22%, of mice transplanted with low, mid, or high cell dose respectively were able to complete the rotarod test (180 sec) while media-injected mice could not complete the test (Fig. 1E). At 17 weeks of age, about 6% of mice receiving the high 1 × 10^6^ cell dose were the only animals to complete the test.

Thus, intravenous transplantation of hBM34+ cells at different doses into symptomatic ALS mice ameliorated behavioral outcomes during 4 weeks post-treatment vs. media-injected mice by maintaining body weight and delaying losses in hindlimb extension, muscle strength, and rotarod performance. However, the most beneficial effect on motor function was determined in G93A mice treated with high 1 × 10^6^ cell dose.

### Spinal cord microvascular permeability

Capillary permeability was examined via quantitative analysis of Evans Blue (EB) extravasation into the spinal cord parenchyma of high cell dose, media-injected, and control mice at 17 weeks of age. Tissue measurements showed significantly higher EB levels in media-injected mice (5.13 ± 0.79 μg/g, p < 0.01) vs. controls (1.52 ± 0.54 μg/g). Significantly (p < 0.01) decreased parenchymal EB level was determined in the spinal cord of mice treated with high cell dose at 4 weeks post-transplant (2.28 ± 0.34 μg/g) compared to media-injected animals. These results might indicate potential BSCB repair in symptomatic ALS mice treated with high 1 × 10^6^ cell dose by prevention of microvascular leakage in the spinal cord.

### Immunohistochemical analysis of administered hBM34+ cells *in vivo*

**Figure 2:**
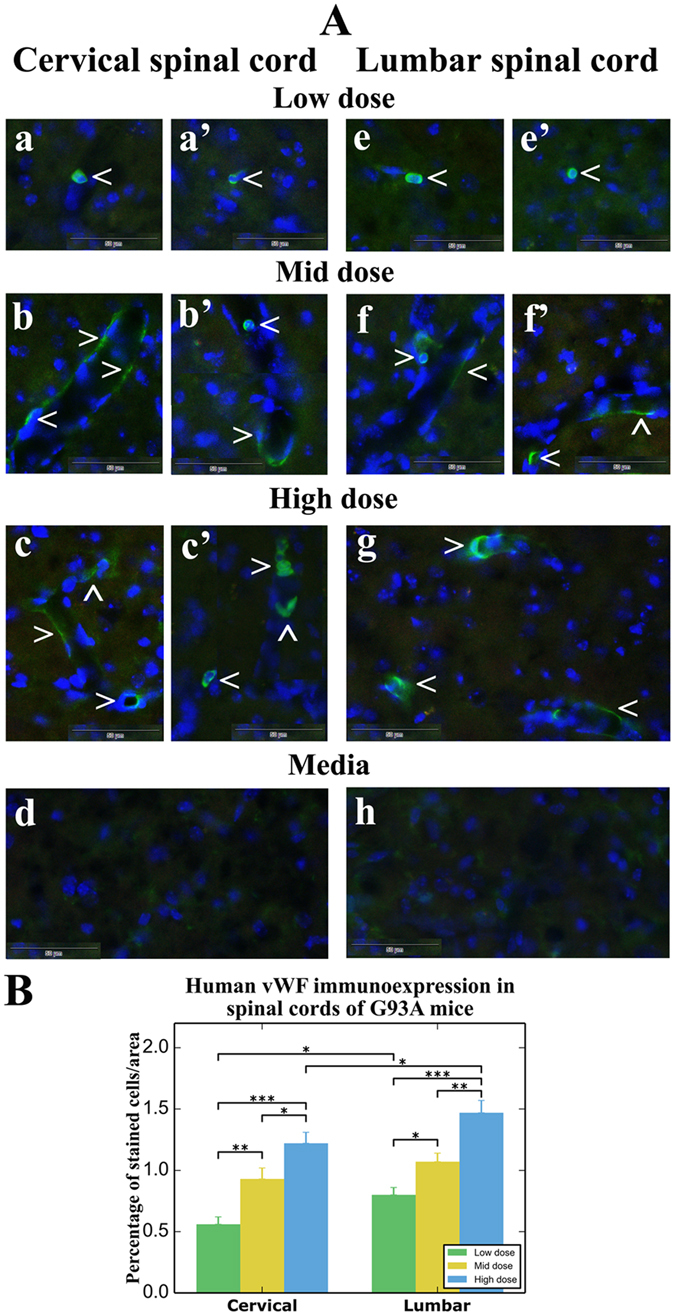
Immunohistochemical staining for human vWF in the cervical and lumbar spinal cords of G93A mice receiving different cell doses. (**A**) Immunopositive vWF expression (green, arrowhead) was indicated in cells with rounded or oval shapes within capillaries of the cervical (a,a’) and lumbar (e,e’) spinal cords of mice receiving the low cell dose. Adherent transplanted cells expressing vWF in lumen of microvessels formed a distinguishable lining in capillary walls (green, arrowhead) in the cervical (b, b’) and lumbar (f,f’) spinal cords of mice with mid cell dose. Numerous capillaries in the cervical (c,c’) and lumbar (g) spinal cords of mice treated with high cell dose showed immunopositive vWF expression (green, arrowhead) indicating impressive amounts of transplanted cells adhering to vascular walls. No immunoexpression of vWF was found in the spinal cords of media-injected mice (d,h). Images are merged with DAPI. Scale bar in a-h is 50 µm. (**B**) Quantitative analysis of fluorescent vWF expression in ventral gray matter of the cervical and lumbar spinal cords showed significant dose-dependent increases in fluorescent expression of vWF. *p < 0.05, **p < 0.01, ***p < 0.001.

Four weeks after intravenous hBM34+ cell transplantation, the cervical and lumbar tissues from mice treated with different cell doses were immunohistochemically stained with human anti-Von Willebrand Factor (vWF), an endothelial cell marker. Cellular vWF immunoexpression was identified mainly in the ventral spinal cord horns of all cell-treated ALS mice. In mice receiving the *low* cell dose, most vWF positive cells were rounded or oval shaped and congregated in capillaries in both cervical (Fig. 2Aa,a’) and lumbar (Fig. 2Ae,e’) spinal cords. Adherence of transplanted cells to the lumen of microvessels by forming a distinguishable line in the capillary walls was determined in ALS mice with *mid* cell dose by vWF immunoexpression in the cervical (Fig. 2Ab,b’) and lumbar (Fig. 2Af,f’) spinal cords. Numerous capillaries in the cervical (Fig. 2Ac,c’) and lumbar (Fig. 2Ag) spinal cords of mice receiving the *high* cell dose showed immunopositive vWF expression in vascular walls, indicating superior adherence of transplanted cells. Positive immunoexpression of vWF was also indicated in dorsal horn and white matter microvessels (data not shown). Of note, cellular vWF immunoexpression was not found in the parenchyma of spinal cords from cell-treated mice or in capillaries/parenchyma from media-treated ALS mice (Fig. 2Ad,h). Quantitative analysis of fluorescent vWF expression in randomly selected areas from right and left ventral gray matter of the cervical and lumbar spinal cords demonstrated significantly increased intensities of vWF fluorescent expression corresponding with higher transplant cell doses (Fig. 2B).

**Figure 3:**
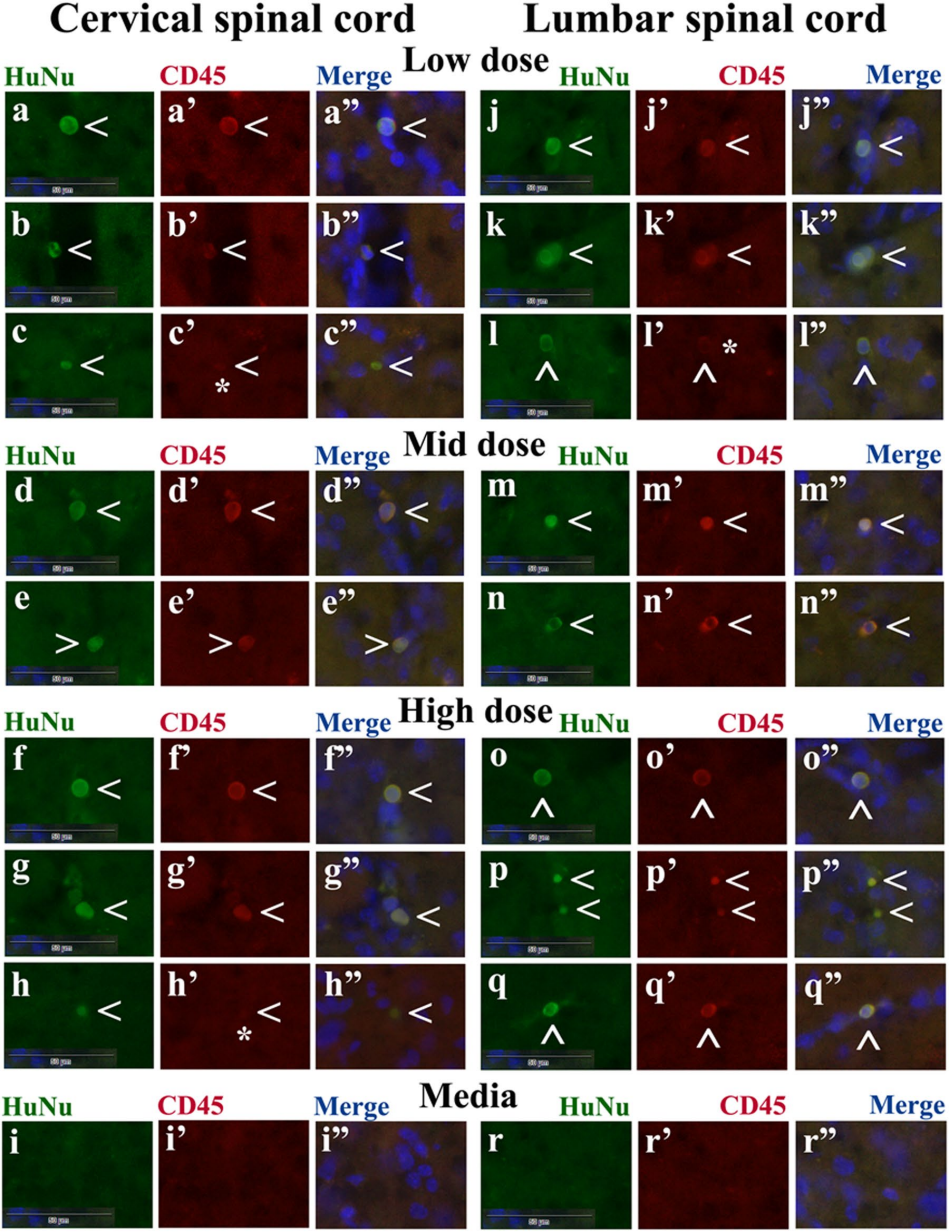
Immunochistochemical staining for HuNu and CD45 in the cervical and lumbar spinal cords of G93A mice receiving different cell doses. Double immunohistochemical staining with human-specific nuclei (HuNu) and CD45 of the cervical spinal cord showed immunopositive cells expressing HuNu+/CD45+ antigens (green, red, arrowhead) in mice treated with low (**a**–b”), mid (**d**–e”), and high (**f**–g”) cell doses. These cells display predominantly rounded morphology and are located within the capillary lumen. A few cells were HuNu+/CD45− (**c**–c”, **h**–h”, asterisk) and are found some distance from blood vessels. Similarly, in the lumbar spinal cords, immunoexpression of HuNu+/CD45+ cells (green, red, arrowhead) was noted in all cell-treated mice: low (**j**–k”), mid (**m**–n”), or high (**o**–q”) cell doses. Some cells only immunoexpressed HuNu and were negative for CD45 (**l**–l”, asterisk). No cells expressing HuNu or CD45 were determined in the cervical (**i**–i”) or lumbar (**r**–r”) spinal cord from media-injected mice. Images are merged with DAPI. Scale bar in (**a**–r”) is 50 µm.

In a separate set of the cervical and lumbar tissues from cell-treated mice, double immunohistochemical staining was performed with the human-specific nuclei (HuNu) and CD45, a hematopoietic common leukocyte marker. In the cervical spinal cord, double immunopositive HuNu+/CD45+ cells were determined in mice treated with low (Fig. 3a–b”), mid (Fig. 3d–e”), and high (Fig. 3f–g”) cell doses. These cells are predominantly of rounded morphology and located within the capillary lumen. A few cells were HuNu+/CD45- (Fig. 3c–c”,h–h”) and were found some distance from blood vessels. Similarly to the cervical spinal cord, immunopositive HuNu+/CD45+ cells were found in the lumbar spinal cords of all cell-treated mice: low (Fig. 3j–k”), mid (Fig. 3m–n”), and high (Fig. 3o–q”) cell doses. Also, some cells only immunoexpressed HuNu and were negative for CD45 (Fig. 3l–l”). No cells expressing HuNu or CD45 were determined in cervical (Fig. 3i–i”) or lumbar (Fig. 3r–r”) spinal cords from media-injected mice.

Thus, immunohistochemical analysis *in vivo* showed that transplanted cells differentiated into endothelial cells and had engrafted into capillaries in the cervical and lumbar spinal cords by 4 weeks post-transplant. However, some administered cells expressed CD45 common leukocyte antigen and some cells were positive only for HuNu, suggesting differentiation of transplanted cells into cells with different immunophenotypes.

### Effect of hBM34+ cell transplantation at different doses on protoplasmic astrocytes

**Figure 4:**
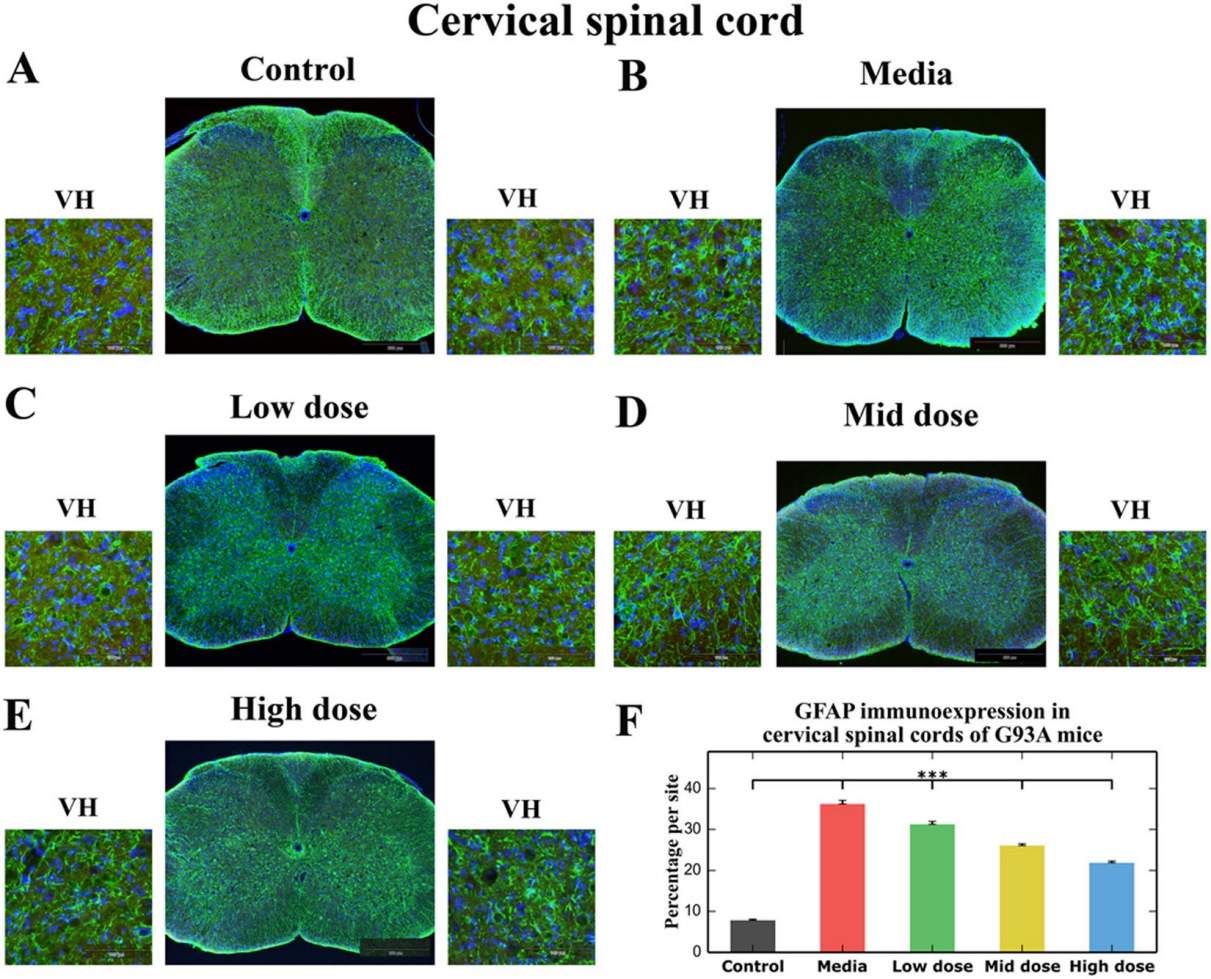
Characteristics of protoplasmic astrocytes in the cervical spinal cord of G93A mice. Immunohistochemical staining of astrocytes using GFAP antibody (green) in (**A**) control mice showed cells with normal cellular morphology distributed relatively uniformly within spinal cord gray matter. (**B**) At 17 wks of age, astrocytes with large cell bodies and hypertrophic processes at high density were determined in media-injected mice. (**C**) Moderate decrease of reactive astrocytes was noted in the ventral horns of ALS mice treated with low cell dose. (**D**) Less astrocyte cell reactivity and more astrocytes with thin cell processes were seen in the gray matter of ALS mice with mid dose. (**E**) Only a few astrocytes with thick cell processes and enormous cell bodies were found in mice after high cell dose transplantation. In these mice, more astrocytes with typical cellular morphology were noted. Scale bar in A-E for full coronal spinal cord section is 500 µm and in ventral horn insert is 100 µm. (**F**) Quantitative analysis of GFAP immunoexpression in the cervical ventral horn demonstrated significantly higher astrocyte density in media-injected mice vs. controls of the same ages. Significant decreases of GFAP immunoexpression were determined in all cell-treated compared to media-injected mice with greatest decrease in mice treated with high cell dose. ***p < 0.001.

Immunohistochemical analysis of astrocytes in the cervical and lumbar spinal cords from cell-treated, media-injected, and control mice at 17 weeks of age was performed by fluorescent immunostaining with GFAP, an intermediate filament protein. In the cervical spinal cord, protoplasmic astrocytes with large cell bodies and hypertrophic processes at high density were determined in media-injected mice (Fig. 4B), while control mice presented astrocytes with fine cellular processes, distributed relatively uniformly within the spinal cord gray matter (Fig. 4A). In ALS mice treated with low cell dose, a moderate decrease of reactive astrocytes was noted in the ventral horns (Fig. 4C). Fewer reactive astrocytes and more astrocytes with thin cell processes were seen in the gray matter of the cervical spinal cords of ALS mice with mid dose (Fig. 4D). Only a few astrocytes with thick cell processes and enormous cell bodies were found in mice after high cell dose transplantation (Fig. 4E). In these mice, more astrocytes with normal morphology were noted. Quantitative analysis of GFAP immunoexpression reflects astrocytic morphology in the cervical ventral horns of analyzed animals and reveals significantly (p < 0.001) higher fluorescent expression of GFAP in media-injected mice (36.21 ± 0.92%) vs. controls (7.83 ± 0.26%) (Fig. 4F). A significant (p < 0.001) decrease of GFAP immunoexpression was determined in all cell-treated compared to media-injected mice. Fluorescent GFAP expressions were inversely proportional to cell doses: low: 31.26 ± 0.71%, mid: 26.05 ± 0.46%, and high: 21.85 ± 0.46% cell doses (Fig. 4F).

**Figure 5:**
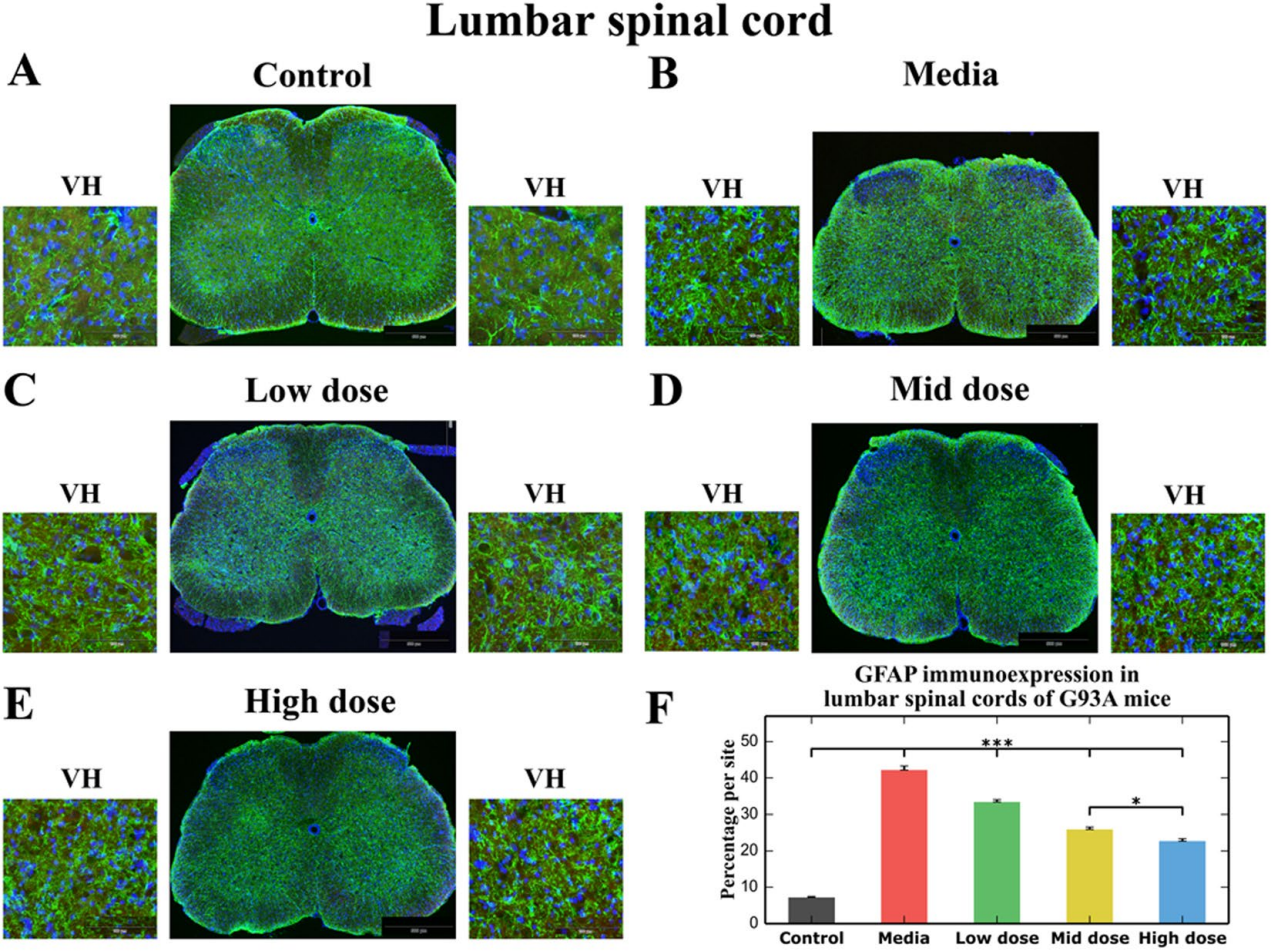
Characteristics of protoplasmic astrocytes in the lumbar spinal cord of G93A mice. Immunohistochemical staining of astrocytes using GFAP antibody (green) in (**A**) control mice showed cells with typical cellular morphology, whereas (**B**) numerous astrocytes with large cell bodies and thick processes were observed in media-injected mice at 17 wks of age. Astrocyte cell reactivity was reduced in the ventral horns of ALS mice treated with low (**C**), mid (**D**), or high (**E**) cell doses. In cell-treated animals, many astrocytes with distinctive fine cell processes at low density were detected. Scale bar in A-E for full coronal spinal cord section is 500 µm and in ventral horn insert is 100 µm. (**F**) GFAP fluorescence in the lumbar ventral horns was significantly upregulated in media-injected mice vs. controls. Significantly decreased GFAP immunoexpression was associated with elevated cell doses. *p < 0.05, ***p < 0.001.

Similarly to the cervical spinal cord, astrocytic morphological profiles in the lumbar spinal cords from cell-treated, media-injected, and control mice at 17 weeks of age were observed. Media-treated ALS mice showed astrocytosis as indicated by protoplasmic astrocytes with large cell bodies and thick processes (Fig. 5B) compared to controls with typical astrocyte morphology (Fig. 5A). In ALS mice treated with low (Fig. 5C), mid (Fig. 5D), or high (Fig. 5E) cell doses, astrocyte cell reactivity was reduced in the ventral horns. Many astrocytes with distinctive fine cell processes at low density were detected in these animals. The measurements of GFAP immunoexpression in the lumbar ventral horns demonstrated a significant (p < 0.001) increase of GFAP fluorescent expression in media-injected mice (42.20 ± 1.09%) versus controls (7.19 ± 0.27%) (Fig. 5F). A significantly (p < 0.001) decreased rate of GFAP immunoexpression was associated with elevated cell doses: low: 33.39 ± 0.66%, mid: 25.90 ± 0.66%, and high: 22.68 ± 0.63% cell doses (Fig. 5F). Of note, the difference between high and mid doses was significant at p < 0.05.

Thus, immunohistochemical analysis of GFAP immunoexpression in the cervical and lumbar spinal cords showed substantial astrogliosis in media-injected mice at 17 weeks of age. In these mice, reactive protoplasmic astrocytes were characterized by large cell bodies and hypertrophic processes. Four weeks after cell transplantation, astrocyte cell reactivity was significantly reduced in cell-treated mice and decreased rates of astrocytosis were associated with elevated cell doses.

### Effect of hBM34+ cell transplantation at different doses on fibrous astrocytes

In the same set of cervical and lumbar spinal cord sections from 17-week-old cell-treated, media-injected, and control mice used for protoplasmic astrocyte analysis, fluorescent images of GFAP immunoexpression of fibrous astrocytes were obtained from lateral and anterior white matter of both sides of the spinal cord at 10X magnification. In the cervical spinal cord, fibrous astrocytes were typically oriented in lateral and anterior white matter in control mice (see Supplementary Fig. 1SA,B). Reactive fibrous astrocytes with hypertrophic processes were mainly determined in the lateral column of media-injected mice. In some areas of anterior white matter, thick astrocyte processes were observed. Fibrous astrocyte reactivity in both lateral and anterior white matter was reduced in cell-treated mice proportionally with elevated cell doses (see Supplementary Fig. 1SA,B). However, some reactive astrocytes were determined in lateral spinal column in mice treated with low or mid cell doses. Similarly to the cervical spinal cord, fibrous astrocytes in analyzed white matter lumbar spinal cord regions of control mice showed normal morphology (see Supplementary Fig. 1SC,D). A substantial increase of GFAP immunoexpression was found in the lateral columns of media-injected mice (see Supplementary Fig. 1SC). Although a lesser degree of GFAP immunoexpression was seen in this white matter area of cell-treated mice, regional density of hypertrophic astrocyte processes was observed. In anterior white matter of the lumbar spinal cord, few thick fibrous astrocyte processes were determined in media-injected and low cell-treated mice (see Supplementary Fig. 1SD).

Thus, GFAP immunoexpression in lateral and anterior white matter in the cervical and lumbar spinal cords showed a substantial increase of reactive fibrous astrocytes in media-injected mice at 17 weeks of age. Four weeks after cell transplantation, reduced astrocyte cell reactivity in areas of white matter tracts in cell-treated mice was associated with increased cell doses.

### Effect of hBM34+ cell transplantation at different doses on perivascular astrocytes

**Figure 6:**
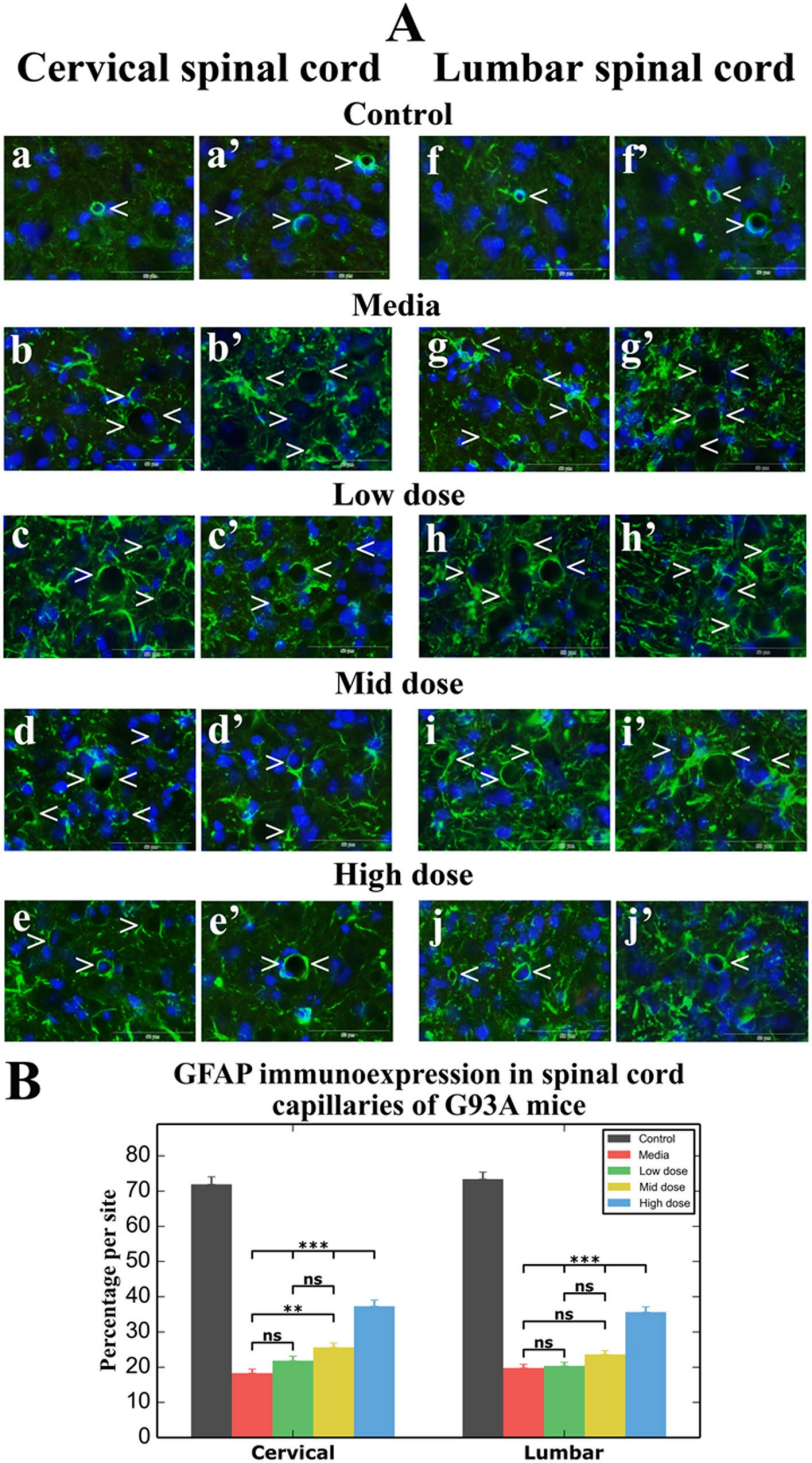
Characteristics of perivascular astrocytes in the cervical and lumbar spinal cord of G93A mice. (**A**) Immunohistochemical staining of perivascular astrocytes with GFAP antibody (green) in control mice showed normal appearance of delineated astrocytes covering capillaries in the cervical (a,a’, arrowheads) and lumbar (f,f’, arrowheads) spinal cords. In media-injected mice, perivascular astrocytes surrounding capillaries were partially revealed in both the cervical (b,b’, arrowheads) and lumbar (g,g’, arrowheads) spinal cords. There were no substantial differences in perivascular astrocyte GFAP expressions in cervical (low dose; c,c’; mid dose: d,d’, arrowheads) or lumbar spinal (low dose; h,h’; mid dose: i,i’, arrowheads) cords compared to media-injected mice. In mice receiving high cell dose, numerous capillaries demonstrated typical perivascular astrocytes surrounding capillaries in both cervical (e, e’, arrowheads) and lumbar (j,j’, arrowheads) spinal cords. Scale bar in a–j’ is 50 µm. (**B**) Measures of GFAP perivascular immunoexpression showed significantly decreased fluorescence in the cervical and lumbar spinal cords of media-injected mice vs. controls. In the cervical spinal cord, significantly increased perivascular GFAP immunoexpressions were determined in mice receiving mid and high cell doses vs. media-injected animals. Also, perivascular GFAP intensity in high cell dose spinal cords was significantly greater compared to low or mid cell doses. In the lumbar spinal cord, no significant differences in perivascular GFAP fluorescent expression were found between media-injected, low, and mid cell doses. Only mice receiving the high cell dose exhibited a significant increase of perivascular immunoexpression vs. media-injected, low, or mid cell dose mouse groups. **p < 0.01, ***p < 0.001.

In the same set of cervical and lumbar spinal cord sections from 17-week-old cell-treated, media-injected, and control mice used for protoplasmic astrocyte analysis, fluorescent images of GFAP immunoexpression of perivascular astrocytes were obtained from ventral horns of both sides of the spinal cord. Normal appearance of delineated perivascular astrocytes fully covering capillaries was observed in the cervical (Fig. 6Aa,a’) and lumbar (Fig. 6Af,f’) spinal cords from control animals. Perivascular astrocytes surrounding capillaries were partially revealed in media-injected mice in both the cervical (Fig. 6Ab,b’) and lumbar (Fig. 6Ag,g’) spinal cords. Also, reactive protoplasmic astrocytes near capillaries were noted in these animals. In mice receiving low or mid cell doses, there were no substantial changes in presence of perivascular astrocytes from cervical (low dose; Fig. 6Ac,c’; mid dose: Fig. 6Ad,d’) or lumbar (low dose; Fig. 6Ah,h’; mid dose: Fig. 6Ai,i’) spinal cords compared to media-injected mice. Only a few capillaries with regularly delineated perivascular astrocytes were observed in spinal cord capillaries from mice receiving the mid cell dose. In contrast, numerous capillaries with typical perivascular astrocytes surrounding capillaries were determined in both cervical (Fig. 6Ae,e’) and lumbar (Fig. 6Aj,j’) spinal cords of high-cell-dose mice. Analysis of fluorescent GFAP perivascular immunoexpression showed similar significant (p < 0.001) decreases of GFAP intensity in both cervical (18.30 ± 1.14%) and lumbar (19.75 ± 1.06%) spinal ventral horns from media-injected mice compared to control mice: cervical: 71.95 ± 2.12% and lumbar: 73.39 ± 1.99% (Fig. 6B). In the cervical spinal cord, significant increases of perivascular GFAP immunoexpression were determined in mice receiving mid (p < 0.01, 25.60 ± 1.26%) and high (p < 0.001, 37.28 ± 1.77%) cell doses vs. media-injected animals. Also, GFAP intensity in high-cell-dose mice was significantly (p < 0.001) greater compared to low or mid cell doses. In the lumbar spinal cord, no significant differences in perivascular GFAP fluorescent expression were found between media-injected (19.75 ± 1.06%), low (20.34 ± 1.04%), and mid (23.60 ± 1.11%) cell doses. Only mice transplanted with high cell dose demonstrated a significant (p < 0.001) increase of perivascular immunoexpression (35.64 ± 1.49%) vs. mice from media-injected, low, or mid cell doses groups (Fig. 6B).

Thus, immunohistochemical analysis of perivascular GFAP immunoexpression showed significant decreases in delineated astrocytes and their perivascular end-feet at the blood capillaries in the cervical and lumbar spinal ventral horns in media-injected mice at 17 weeks of age. Four weeks after cell transplantation, perivascular astrocytes were re-established in numerous capillaries, mainly in mice treated with high cell dose in both the cervical and lumbar spinal cords. Also, mice receiving mid cell dose showed significantly increased perivascular GFAP immunoexpression only in the cervical spinal cords vs. media-injected animals.

### Effect of hBM34+ cell transplantation at different doses on microglia

**Figure 7:**
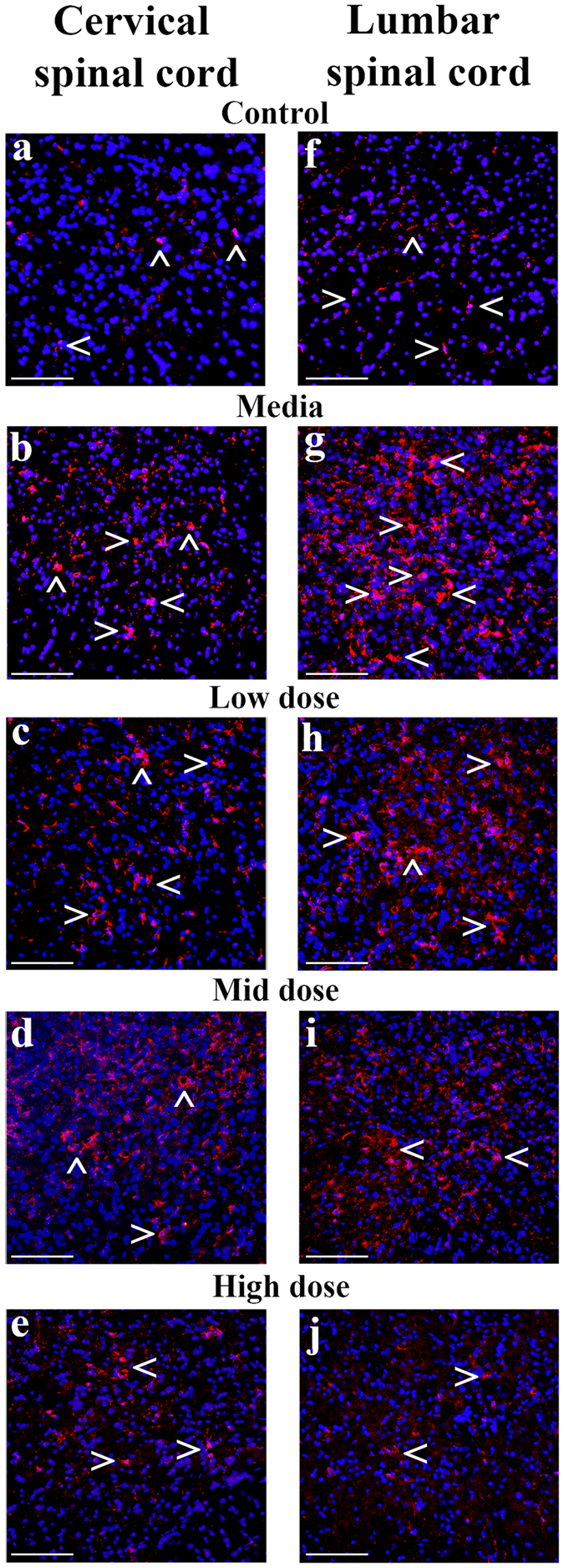
Characteristics of microglial cells in the ventral horn of spinal cords from G93A mice. Immunohistochemical staining of microglia using anti-Iba-1 antibody was performed in the cervical and lumbar spinal cords from cell-treated, media-injected, and control mice at 17 weeks of age. Microglial cells showed typical morphology (red, arrowheads) in the ventral horn of the cervical (**a**) and lumbar (**f**) spinal cords of control mice. Substantial cervical (**b**) and lumbar (**g**) microgliosis was noted in media-treated animals. Morphologically, numerous activated microglial cell with large cellular bodies and short processes were observed (arrowheads). Microglial cell activation was decreased in the cervical and lumbar ventral horns of ALS mice treated with low (**c**,**h**), mid (**d**,**i**), or high (**e**,**j**) cell doses. In cell-treated animals, many microglial cells with smaller cell bodies and fine cell processes (arrowheads) at a low density of Iba-1 immunoexpression were detected. Ramified microglia were mostly observed in the cervical and lumbar spinal cords of mice receiving the high cell dose. Scale bar in (**a**–**j**) is 100 µm.

Immunohistochemical analysis of microglia in the cervical and lumbar spinal cords from cell-treated, media-injected, and control mice at 17 weeks of age was performed by fluorescent immunostaining with anti-Iba-1 antibody. In the cervical spinal cord, microglial cells with large cell bodies and thick processes at high density were determined in the ventral horn of media-injected mice (Fig. 7b) compared to control mice presenting a few microglia with fine cellular processes (Fig. 7a). In ALS mice treated with low (Fig. 7c) or mid (Fig. 7d) cell doses, a moderate decrease of Iba-1 immunoexpression was noted in the ventral horns. Fewer activated microglial cells, determined morphologically by large cell bodies and short processes, were seen in the ventral horn of the cervical spinal cords of these treated ALS mice. In mice receiving the high cell dose, more microglia with normal cellular morphology were found (Fig. 7e).

Similarly to the cervical spinal cord, microglial morphological profiles in the lumbar spinal cords from cell-treated, media-injected, and control mice at 17 weeks of age were determined. Media-treated ALS mice showed massive microgliosis in the ventral horn, as indicated by numerous microglial cells with large cell bodies and thick processes (Fig. 7g), compared to controls with typical cell morphology (Fig. 7f). Of note, more activated microglial cells were observed in the lumbar spinal cord vs. cervical in media-injected mice. In ALS mice treated with low (Fig. 7h), mid (Fig. 7i), or high (Fig. 7j) cell doses, microglial cell activation was reduced in the ventral horns. Many ramified cells with smaller cell bodies and fine cell processes at low density were detected in these animals. Particularly, mice receiving high cell dose showed Iba-1 immunoexpression in the ventral horn of the lumbar spinal cord near levels detected in control animals.

Thus, immunohistochemical analysis of Iba-1 immunoexpression in the cervical and lumbar spinal cords showed substantial microgliosis in media-injected mice at 17 weeks of age. In these mice, activated microglial cells were characterized by large cell bodies and thick processes. Four weeks after cell transplantation, microglial cell activation was significantly reduced in cell-treated mice and decreased rates of microgliosis were associated with elevated cell doses.

### Effect of hBM34+ cell transplantation at different doses on motor neuron survival

A separate set of cervical and lumbar spinal cord sections from randomly selected cell-treated, media-injected, and control mice was stained with 0.1% cresyl violet for examination of motor neuron condition. Histological and stereological analyses of motor neurons in the ventral horn of the cervical and lumbar spinal cords were performed at 17 weeks of age.

**Figure 8:**
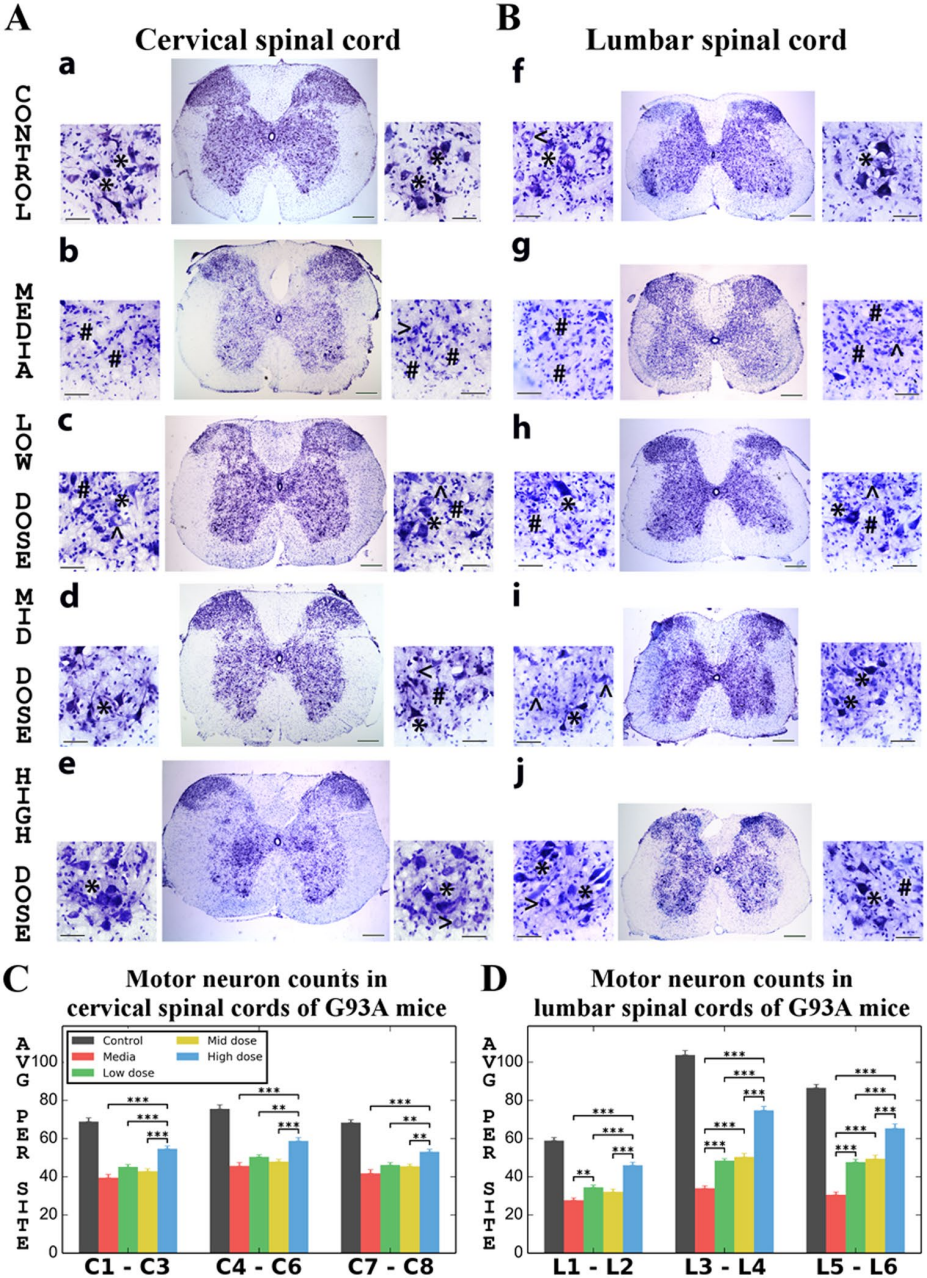
Characteristics of motor neurons in the cervical and lumbar spinal cord of G93A. Histological analysis of motor neurons in (**A**) cervical (C3) and (**B**) lumbar (L3) spinal cords (cresyl violet staining) showed healthy motor neurons in control animals (a,f, asterisks). Most motor neurons had degenerated or vacuolated in the cervical (b) and lumbar (g) spinal cords of media-injected mice at 17 wks of age. Mice receiving low or mid cell dose demonstrated more healthy-appearing motor neurons (asterisks) in both segments of the spinal cord (low dose – c,h; mid dose – d,i); yet some degenerated motor neurons were noted in addition to vacuolated cells. Higher motor neuron survival in the cervical (e) and lumbar (j) spinal cords was determined in mice treated with high cell dose. Only a few motor neurons were vacuolated. * - healthy motor neuron, # - degenerated motor neuron, < - vacuolated motor neuron. Scale bar in a-j for full coronal spinal cord section is 200 µm and in ventral horn insert is 50 µm. (**C**) Stereological motor neuron counts in discrete levels of the cervical spinal cord showed significantly decreased motor neuron survival in media-injected mice vs. controls. Only ALS mice receiving the high cell dose demonstrated significantly greater motor neuron numbers in all analyzed spinal cord levels compare to other cell-treated or media-injected mice. (**B**) In the lumbar spinal cord, significantly lower motor neuron numbers at all discrete cord levels were found in media-injected mice vs. controls, similar to results in the cervical spinal cord. Stereological counts of motor neurons in mice treated with different cell doses showed significantly increased cell numbers in the ventral horns compared to media-injected animals. Best motor neuron survival was determined in mice treated with high cell dose. **p < 0.01, ***p < 0.001.

In control mice, healthy motor neurons with large soma and neuritic processes were determined in both cervical (Fig. 8Aa) and lumbar (Fig. 8Bf) spinal cords. In media-injected mice, most motor neurons had degenerated or vacuolated (Fig. 8Ab,Bg). Only a few healthy motor neurons were identified in the ventral horn of these mice. ALS mice receiving low (Fig. 8Ac,Bh) or mid (Fig. 8Ad,Bi) cell doses demonstrated robust appearing motor neurons in both segments of the spinal cord vs. media-injected mice. However, some degenerated motor neurons were visible. Also, differently sized motor neurons displayed vacuolization in these cell-treated mice. In contrast, many healthy motor neurons with large soma were determined in the cervical (Fig. 8Ae) and lumbar (Fig. 8Aj) spinal cords in mice receiving the high cell dose. Only a small number of motor neurons were vacuolated.

Stereological motor neuron counts were performed in discrete segmental levels in the ventral horns of cervical (C1–C3, C4–C6, and C7–C8) and lumbar (L1–L2, L3–L4, and L5–L6) spinal cords. Analysis demonstrated a significant (p < 0.001) decrease of motor neuron survival in media-injected mice vs. controls at all analyzed spinal cord levels (Fig. 8C,D). In the cervical spinal cord, only mice receiving the high cell dose showed significantly (p < 0.001 or p < 0.01) higher motor neuron numbers compare to other cell-treated groups or media-injected mice (Fig. 8C). More surviving motor neurons were found at the enlarged C4–C6 cervical cord level in these animals (58.94 ± 1.67 number/side) vs. low (50.46 ± 1.11 number/side), mid (47.97 ± 1.26 number/side) cell doses, or media-injected (45.67 ± 1.84 number/side) mice.

Similarly, significantly (p < 0.001) lower motor neurons counts were determined in lumbar spinal cords of media-injected mice vs. controls (Fig. 8B). ALS mice receiving low dose, mid dose, or high cell doses presented significantly (at different confidence levels of p < 0.001 or p < 0.01) greater motor neuron survival in all discrete segmental levels compared to media-injected animals. Also, motor neuron numbers in mice receiving the high cell dose were superior (p < 0.001), mainly in the enlarged lumbar level at L3–L4, compared to low or mid cell doses. Motor neuron numbers in the lumbar spinal cord at L3–L4 level in G93A mice at 17 weeks of age were: low dose – 48.43 ± 1.10, mid dose – 50.42 ± 1.87, high dose – 74.84 ± 2.10, and media – 34.04 ± 1.26 neurons/side (Fig. 8B).

Thus, histological and stereological analyses of motor neuron staining for the Nissl substance in the ventral horn of the cervical and lumbar spinal cords demonstrated significant losses of motor neurons in media-injected mice at 17 weeks of age. Although some robust appearing motor neurons were indicated in spinal cords of mice with low or mid cell dose treatment, superior motor neuron survival was determined in cervical and lumbar spinal cords from mice receiving high cell dose at four weeks after transplantation.

## Discussion

In the present study, the therapeutic effects of intravenously transplanting different doses of unmodified human bone marrow CD34^+^ (hBM34+) cells into symptomatic G93A SOD1 mice were evaluated in the context of potential mechanisms of BSCB repair. The major study findings were that the high dose of hBM34+ cells effectively: (1) improved behavioral disease outcomes; (2) differentiated into endothelial cells and engrafted into spinal cord capillaries; (3) decreased EB extravasation into spinal cord parenchyma; (4) reduced astrogliosis and microgliosis; (5) improved perivascular end-feet astrocyte integrity; and (6) enhanced spinal cord motor neuron survival. Although low (5 × 10^4^ cells) and mid (5 × 10^5^ cells) cell doses demonstrated some of the above benefits, the high dose of 1 × 10^6^ cells proved most beneficial, indicating the importance of cell dosage in advanced stage disease. Here, we are the first to demonstrate, from a translational viewpoint, benefits of unmodified hematopoietic stem cells derived from bone marrow when treatment was initiated at the symptomatic disease stage, potentially leading to BSCB restoration in ALS. Specifically, differentiation of hBM34+ cells into endothelial cells and their engraftment into spinal cord capillaries as well as reduction of EB spinal cord extravasation and enhancement of perivascular end-feet astrocyte capillary coverage at 4 weeks post-transplantation were detected as potential regenerative processes towards BSCB repair in ALS.

Cell-based therapy to modify the pathological microenvironment and thus promote motor neuron survival has been proposed as a therapeutic strategy for ALS^55–57^. Stem cells are known to secrete various growth or trophic factors that may modulate the local microenvironment to rescue diseased motor neurons^58–64^. However, repairing the B-CNS-B to restrict entry of detrimental factors from the systemic circulation to the CNS may be a new, more feasible, yet underexplored therapeutic approach for motor neuron survival in ALS. Barrier restoration may be achieved by replacement of damaged endothelial cells and could potentially lead to improvements in disease outcomes.

Our study results showed that intravenous transplantation of hBM34+ cells at different doses into symptomatic ALS mice ameliorates neurobehavioral dysfunction during 4 weeks post-treatment vs. media-injected mice. Yet, the most beneficial effects on motor function were determined in G93A mice treated with 1 × 10^6^ cells. Some of these animals (6%) were able to fully complete the rotarod test at 17 weeks of age, typically end-stage of disease. Post-transplant motor function improvements, mainly in mice receiving the high cell dose, indicate delayed disease progression and are supported by observations of motor neuron survival in the spinal cord.

Our histological and stereological analyses of motor neurons in discrete segmental levels in the ventral horn of the spinal cords at 17 weeks of age demonstrated superior motor neuron survival mostly in mice receiving the high cell dose. More surviving motor neurons in these animals were noted in enlarged segments of the cervical (C4–C6) and lumbar (L3–L4) spinal cords compared to other cell-treated or media-injected mice. For instance, mice receiving the high 1 × 10^6^ cell dose showed more than a two-fold increase in motor neuron numbers at L3–L4 segments vs. media-injected. Also, some healthy-appearing motor neurons were indicated in the spinal cords of mice with low or mid cell dose treatment. The four distinguished stages of disease progression in G93A SOD1 mice: pre-symptomatic (30–60 days), symptomatic (100 days, approximately 50% loss of motor neurons and function), end-stage of disease (120 days, near complete hindlimb paralysis), and death (about 4–5 months of age), are well documented and widely used in investigations of disease pathogenesis^4, 65–70^ and treatment efficacy. Motor function impairment in G93A SOD1 mice is first detectable in the hindlimbs, which are innervated by the lumbar spinal cord, and later motor weakness is seen in the forelimbs, innervated by the cervical spinal cord^4^. This differential involvement of the forelimbs and hindlimbs during disease progression is consistent with our study results showing higher motor neuron numbers in the ventral horn of cervical vs. lumbar spinal cord in media-injected mice at 17 weeks of age. However, discrete segmental level analysis of motor neurons in the ventral horn of the spinal cords in our study not only determined cervical or lumbar spinal cord areas affected by disease (non-treated mice), but was also imperative to determine cell-transplant effects on motor neuron survival in distinct segments of the spinal cord in mice. To our knowledge, such motor neuron analysis in discrete spinal cord levels has not been reported previously. However, since the present study was primarily focused on disease progression in G93A mice by determining behavioral disease outcomes during 4 weeks post-transplant and motor neuron survival at 17 weeks of age, evaluating the effect of cell treatment on mouse lifespan is an important next step. This study is currently underway.

Motor function improvements in cell-treated ALS mice concurrent with enhanced motor neuron survival were likely achieved by transplanted cell differentiation. Our *in vivo* immunohistochemical analysis showed differentiation of transplanted cells into endothelial cells as determined by human vWF immnoexpression and engraftment of these endothelial cells into capillaries in the cervical and lumbar spinal cords at 4 weeks post-transplantation. Although administered cells expressing vWF antigen were identified in all cell-treated ALS mice, mainly in the ventral spinal cord horns, mice receiving the low cell dose showed that most vWF positive cells congregated within capillaries without attaching to vessel walls in both cervical and lumbar spinal cords. However, mice receiving mid or high cell doses displayed transplanted cell adherence in lumen of microvessels forming a distinguishable lining in the capillary walls in the spinal cords. More capillaries demonstrating adherence of transplanted cells to vascular walls in the cervical and lumbar spinal cords were determined in mice receiving the high cell dose. This novel finding indicates that transplantation of hBM34+ cells at an optimal cell dose into symptomatic ALS mice may restore the damaged BSCB by endothelial cell differentiation and capillary integration. This suggestion was prompted by the significant reduction of EB extravasation into spinal cord parenchyma in mice treated with the high cell dose. However, the effects of low and mid cell doses on capillary permeability in the spinal cord should be determined and are presently under examination. Of note, our data showing a high level of EB leakage in the spinal cords of media-injected ALS mice confirmed a previous finding in symptomatic SOD1-linked ALS rats^29^. However, some transplanted cells expressed CD45 and some cells were positive only for HuNu marker, representing potential differentiation of administered cells into cells with different immunophenotypes. These data led to the suggestion that transplanted hBM34+ cells have the potential to provide beneficial effects on motor neurons survival by additional cell actions other than those promoting BSCB repair in ALS.

One limitation of this study is that BSCB status is not definitively examined in cell-treated animals; various characteristics of BSCB integrity are currently under investigation. These studies are primarily designed to determine cellular (endothelial and pericyte) reliability and capillary basement membrane consistency in ALS mice treated with different cell doses vs. non-treated animals. In addition to endothelial cell status, a specific focus will be pericytes due to their critical role for B-CNS-B maintenance by pericyte-endothelial cell signal transduction and close proximity^71–75^. It has been shown that capillary coverage and pericyte numbers are reduced in the regional spinal areas compared to brain regions in wild-type mice^76^. This regional reduction in spinal cord pericytes was associated with increased capillary permeability and decreased tight junction protein expressions. Furthermore, BSCB disruption leading to serum protein leakage and motor neuron loss was determined in pericyte-deficient mutant mice, confirming the importance of this cellular constituent for BSCB integrity^76^. Also, a recent study demonstrated that loss-of-pericyte-function in pericyte-deficient mice leads to reduced cerebral capillary blood flow resulting in neurovascular uncoupling and limiting oxygen supply to the brain^77^. However, since degenerated pericytes in addition to perivascular collagen IV expansion have only been identified in post-mortem brain and spinal cord tissues of ALS patients (reviewed in ref. 28), capillary pericyte number/coverage in not-treated symptomatic ALS mice are currently being re-examined prior to evaluation of this cellular BSCB component in cell-treated mice.

Since CD34^+^ cells are pluripotent hematopoietic stem cells able to differentiate into multiple hematopoietic cell lineages, commitment to multipotential cell lineages are likely regulated by various intrinsic properties^44^ and microenvironmental factors. It has been shown that bone marrow hematopoietic stem cells transdifferentiate into cells expressing neuronal specific antigens in the brain after intraperitoneal transplantation into mice, which lacked development of the myeloid and lymphoid cell lineages^78^, or hepatocytes after intravenous transplantation into a mouse model of fatal tyrosinemia type 1^79^. When bone marrow-derived CD34^+^ cells are transplanted into patients with ischemic or degenerative retinal conditions^45^ or cardiomyopathy^46^, administered cells migrate into damaged tissue and have been implicated in tissue repair via revascularization through formation of new blood vessels from existing vascularity in ischemic tissues.

In an initial report by Appel and colleagues^80^, allogeneic mobilized CD34^+^ peripheral blood cells were intravenously infused into irradiated SALS patients. Results showed that the treatment was well tolerated by patients, with no treatment-related deaths or toxicities. Although no clinical benefits were noted, all patients demonstrated mixed chimeras at day 30 of treatment. Analysis of autopsied brain and spinal cord tissues revealed 100% engraftment of donor-derived DNA in brainstem, spinal cord, and motor cortex from some patients using RT-PCR analysis. Also, immunohistochemical analysis showed increased CD68 (macrophage-monocyte marker) or CD1-a (dendritic cell marker) positive cells in spinal and brain tissues, indicating that engrafted cells are acting as immunomodulatory cells. The importance of these study findings is showing migration of administered CD34^+^ cells into “the human CNS primarily at sites of motoneuron pathology”^80^ and the potential of these cells to modulate immune/inflammatory response in ALS pathological microenvironment.

Accumulating evidence has demonstrated involvement of the immune/inflammatory system in ALS pathogenesis^19–21, 23, 24, 66, 68, 70, 81–84^. Progressively large numbers of activated microglia and reactive astrocytes, IgG, and T lymphocytes, which play key roles in the inflammatory reaction, were determined in the brainstem and spinal cord of both ALS patient and animal models^66, 82, 85–89^. Reactive astrocytes and activated microglia were detected in cervical and lumbar spinal cord gray matter of G93A mice before or at disease onset and they progressively increased until end-stage of disease^66, 68, 82, 90, 91^. These glial cells have been identified as major inflammatory effectors contributing to motor neuron damage^66, 92–96^ by secretion of various pro-inflammatory cytokines^86–88^. Reactive astrocytosis is directly involved in motor neuron death, while activated microglia aggravate local inflammation during disease progression^97, 98^. However, since BSCB impairment was found in rodent models of ALS prior to evidence of motor neuron degeneration and neuroinflammation^29–31^, inflammatory response in ALS is likely triggered by B-CNS-B damage^33^. Moreover, barrier disruption causing appearance of microhemorrhages with associated release of neurotoxic hemoglobin-derived products from extravasated erythrocytes into CNS parenchyma of SOD1 transgenic mice^30, 31^ might also exacerbate neuroinflammation. Similarly, perivascular deposits of erythrocyte-derived hemoglobin and hemosiderin as well as spinal cord parenchymal accumulation of plasma-derived IgG, fibrin, and thrombin were determined in post-mortem tissues from ALS patients^27, 33^.

There are nine defined classes of astrocytes within the rodent CNS based on cell specific protein expressions, distinct morphologies, regional locations, and specified functions^99, 100^. The particular interest in this study was evaluating status of protoplasmic, fibrous, and perivascular end-feet astrocytes in the spinal cords of G93A mice. As expected, immunohistochemical analysis of GFAP immunoexpression showed substantial astrogliosis in the cervical and lumbar spinal cords in media-injected mice at 17 weeks of age. In these mice, a high density of reactive protoplasmic astrocytes with large cell bodies and hypertrophic processes was determined in the ventral horns. However, astrocyte cell reactivity was significantly reduced in cell-treated mice, and decreased rates of astrocytosis were associated with elevated cell dose treatments at 4 weeks post-transplantation. Additionally, reactive fibrous astrocytes with hypertrophic processes were mainly determined in lateral and in some anterior white matter areas of the cervical and lumbar spinal cords from media-injected mice. Observed astrocyte reactivity in areas of white matter tracts of non-treated symptomatic ALS mice might indicate diminishing of ascending and descending spinal cord tracts. According to reported location of these tracts in mice^101^, our studies mainly detected astrogliosis in regions of rostral/caudal reticulospinal and rubrospinal descending tracts in addition to dorsolateral/lateral/anterior spinothalamic ascending tracts. Degeneration of white matter tracts has been shown to be widespread, from the ventral to dorsolateral funiculi, in G93A SOD1 mice beginning at 16 weeks of age due to axonal pathology^102^. Progressive loss of corticospinal, bulbospinal, and rubrospinal neurons involves the regression of descending corticospinal, bulbospinal, and rubrospinal tracts^103^. However, whether reactive astrocytes are affecting or reflecting deterioration of spinal cord pathways is still unclear. Performing combined anterograde or retrograde tracing methods with GFAP immunoexpression might define the role of astrocytes in diminishing white matter tracts in ALS mice. Also, determination of axonal myelination in non-treated and cell-treated ALS mice is needed to confirm status of the spinal cord tracts. Besides these suggested studies, reduction of fibrous astrocyte reactivity in both lateral and anterior white matter of the cervical and lumbar spinal cords was observed after 4 weeks of treatment in cell-treated mice proportionate to elevated cell doses. However, some regional density of hypertrophic astrocyte processes was seen mostly in the lumbar spinal cords of these mice. Additionally, substantial microgliosis, as indicated by appearance of numerous activated microglial cells with large bodies and short processes, was detected in the cervical and lumbar spinal cords of media-injected mice at 17 weeks of age. Similar to reduction of astrocytosis in the spinal cords of cell-treated mice, microglial cell activation also decreased in spinal ventral horns in these mice consequent to increased cell treatment doses. Thus, inhibition of protoplasmic and fibrous astrocyte reactivity in addition to decrease of activated microglia by hBM34+ cell treatment, mainly by the high cell dose, indicate that reduction of inflammatory responses enhanced by macro- and microgliosis might have contributed to the observed protection of motor neurons and white matter spinal tracts.

Finally, our immunohistochemical analysis of perivascular GFAP immunoexpression showed significant decreases in delineated astrocytes and their perivascular end-feet at the blood capillaries in the cervical and lumbar spinal ventral horns in media-injected mice at 17 weeks of age. These data support our previous findings^25, 26^ demonstrating degeneration of astrocyte end-feet processes surrounding microvessels among endothelial cells in motor neuron areas within the cervical and lumbar spinal cords and brainstem in G93A mice at early and late stages of disease. Progressive astrocyte degeneration leading to the formation of protein-filled areas (edema) in the extravascular space formely occupied by perivascular astrocytes or neuropil provided additional evidence of B-CNS-B disintegration in symptomatic ALS mice^25^. Astrocytes are essential components of the neurovascular unit, providing a functional link between microvessel endothelium and neurons (reviewed in ref. 104). The processes of protoplasmic astrocytes closely contact the outer surface of blood microvessels, forming perivascular end-feet, as well as establishing multiple neuronal contacts. Typically, delineated astrocytes and their perivascular end-feet processes fully cover the capillary surface for proper maintainenance of BBB/BSCB integrity by controlling exchanges of water and solutes between blood and CNS tissue^105, 106^. Therefore, perivascular end-feet astrocytes can influence BBB/BSCB status under both physiological and pathological conditions. In the present study, perivascular end-feet astrocytes were re-established in numerous capillaries in the cervical and lumbar spinal cords, primarily in mice treated with the high cell dose at 4 weeks after transplantation. This major finding suggests that dampening of inflammation propelled restoration of the neurovascular unit. However, anti-inflammatory cell transplant effects need to be elucidated. Potentially, transplanted cells could secrete growth or trophic factors upon their differentiation and thus modulate the pathological microenvironment in ALS. We are planning a study in the near future to investigate this possibility.

Additionally, pharmacological intervention alone or/and in combination with cell therapeutic approach for modulation of neuroinflammation in ALS might be achievable. One potential treatment drug is activated protein C (APC), an endogenous plasma protease with anti-thrombotic, cytoprotective, and anti-inflammatory activities in the CNS^107–111^. Targeting the “vasculo-neuronal-inflammatory triad” as a pathological trio in CNS injuries and disorders by multiple-action-multiple-target agents such as APC has been proposed for potential disease-modifying therapies (reviewed in ref. 112). This attractive approach might lead to stabilization of the BBB/BSCB, neuroprotection and control of neuroinflammation. Moreover, it has been shown that APC can across the BBB to achieve favorable concentrations in the CNS and this transport is mediated by the endothelial protein C receptor^113^. In a study by Zhong *et al*.^114^, daily intraperitoneal injections of APC analogs into G93A SOD1 mice after disease onset slowed disease progression and extended lifespan. Also, APC reduced leakage of hemoglobin-derived products across the BSCB and delayed microglia activation, suggesting that barrier repair was accompanied by anti-inflammatory drug action. Similarly, treatment with an APC analog of spontaneous or warfarin-accelerated microvascular lesions in G93A SOD1 mice demonstrated restoration of BSCB integrity, leading to decreases of neurotoxic hemoglobin and iron deposits^115^. However, although APC treatment showed beneficial effect on reducing barrier leakage, a potential complication to clinical approach of APC for ALS is increased risk of bleeding (hemorrhages)^109^.

In conclusion, intravenous administration of an optimal dose of unmodified bone marrow-derived hematopoietic stem cells into symptomatic ALS mice leads to delayed disease progression as confirmed by improvements in motor function and motor neuron survival. These beneficial functional outcomes might have been engendered by dual cell actions. First, transplanted cells differentiate into endothelial cells and engraft into capillary walls in the spinal cord leading towards BSCB restoration. Also, the significant decrease of capillary permeability in the spinal cord strongly supports our suggestion. Second, transplanted cells potentially modulate the inflammatory microenvironment in the spinal cord by attenuating astrogliosis, microgliosis, and enhancing perivascular end-feet eminence. These potential cell actions might have dependently or independently supported motor neuron survival. Therefore, endothelial cell differentiation, capillary grafting, reduction of capillary permeability, and re-establishment of perivascular end-feet astrocytes through hBM34+ cell transplantation of optimal cell dose into symptomatic ALS mice pose as robust regenerative mechanisms towards BSCB repair, lending translational research support to the utility of hBM34+ cell transplantation as a future therapeutic strategy for ALS patients.

## Electronic supplementary material

Supplementary Figure 1S
