# Supplementary material for: Endothelial and Astrocytic Support by Human Bone Marrow Stem Cell Grafts into Symptomatic ALS Mice towards Blood-Spinal Cord Barrier Repair
Source: Sci Rep. 2017 Apr 13;7:884. doi: 10.1038/s41598-017-00993-0 (PMC5429840; doi:10.1038/s41598-017-00993-0)
Supplement: Supplementary file 1 — Supplementary Figure 1S [file 41598_2017_993_MOESM1_ESM.pdf]

Endothelial and Astrocytic Support by Human Bone Marrow Stem Cell Grafts into Symptomatic ALS Mice towards Blood-Spinal Cord Barrier Repair

Svitlana Garbuzova-Davis, Crupa Kurien, Avery Thomson, Dimitri Falco, Sohaib Ahmad, Joseph Staffetti, George Steiner, Sophia Abraham, Greeshma James, Ajay Mahendrasah, Paul R. Sanberg, Cesario V. Borlongan

Characteristics of fibrous astrocytes in the spinal cord white matter of G93A mice

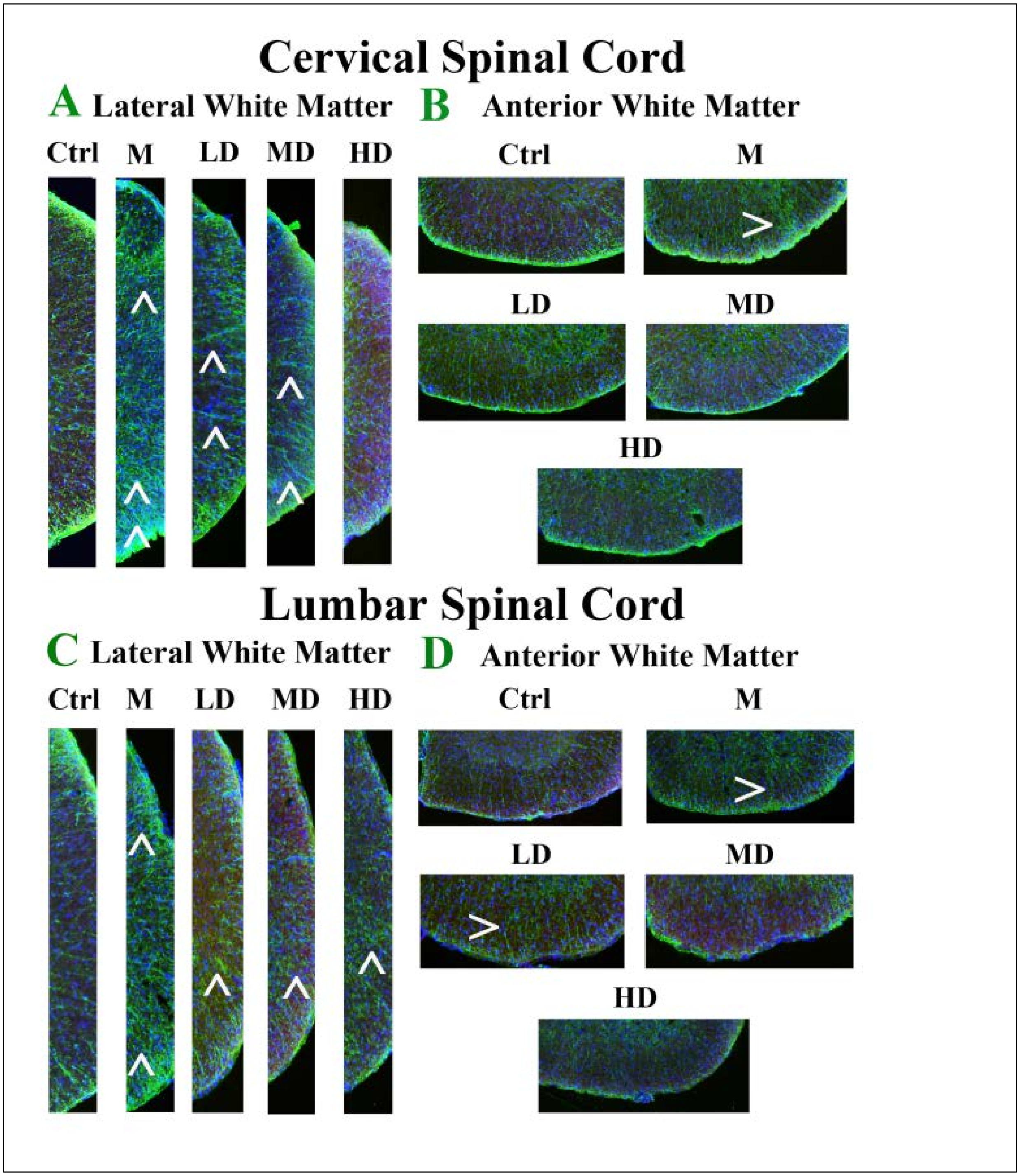

Supplementary Figure 1S. In the cervical spinal cord, immunohistochemical staining of fibrous astrocytes using GFAP antibody (green) in control mice showed cells with typical appearance and orientation in lateral (**A**) and anterior (**B**) white matter. At 17 wks of age, reactive fibrous astrocytes with hypertrophic processes (arrowheads) were mainly determined in lateral column of media-injected mice. In some areas of anterior white matter, thick astrocyte processes (arrowhead) were observed. Fibrous astrocyte reactivity in both lateral (**A**) and anterior (**B**) white matter was reduced in cell-treated mice proportionally with elevated cell doses; yet, some reactive astrocytes (arrowheads) were seen in lateral column in mice receiving low or mid cell doses. In the lumbar spinal cord, fibrous astrocytes in lateral (**C**) and anterior (**D**) white matter regions of control mice showed normal morphology. Substantial increase of GFAP immunoexpression (arrowheads) was found in lateral column of media mice. Although less astrocyte cell reactivity was seen in this white matter area of cell-treated mice, regional density of hypertrophic astrocyte processes (arrowheads) was observed in these mice. In anterior white matter of the lumbar spinal cord, few thick fibrous astrocyte processes (arrowhead) were determined in media and low cell-treated mice. Magnification in all images is 10X. Ctrl – control, M – media, LD – low cell dose, MD – mid cell dose, HD – high cell dose.
